# OATP1B-type Transport Function Is a Determinant of Aromatase Inhibitor–Associated Arthralgia Susceptibility

**DOI:** 10.1158/2767-9764.CRC-24-0475

**Published:** 2025-03-27

**Authors:** Hanieh Taheri, Yang Li, Kevin M. Huang, Eman Ahmed, Yan Jin, Thomas Drabison, Yan Yang, Samuel K. Kulp, Nicholas A. Young, Junan Li, Xiaolin Cheng, Kara N. Corps, Christopher C. Coss, Jennifer E. Vaughn, Maryam B. Lustberg, Alex Sparreboom, Shuiying Hu

**Affiliations:** 1 Division of Pharmaceutics and Pharmacology, College of Pharmacy, The Ohio State University, Columbus, Ohio.; 2 Division of Medicinal Chemistry and Pharmacognosy, College of Pharmacy, The Ohio State University, Columbus, Ohio.; 3 Division of Rheumatology and Immunology, Department of Internal Medicine, The Ohio State University Wexner Medical Center, Columbus, Ohio.; 4 Department of Veterinary Biosciences, College of Veterinary Medicine, The Ohio State University, Columbus, Ohio.; 5 Division of Hematology, Department of Internal Medicine, The Ohio State University, Columbus, Ohio.; 6 Yale Comprehensive Cancer Center, Yale School of Medicine, New Haven, Connecticut.

## Abstract

Aromatase inhibitors (AI) such as anastrozole, letrozole, and exemestane are used as adjuvant treatment for postmenopausal women with hormone receptor–positive breast cancer. The interindividual pharmacokinetic variability seen with AIs is extensive, and this phenomenon may have important ramification for AI-associated arthralgia, a common toxicity of which the etiology remains unclear. We speculated that hepatic uptake transporters involved in the elimination of AIs play a crucial role in explaining this pharmacologic variability. Using an array of *in silico*, *in vitro*, *in vivo*, and human studies, we identified OATP1B1 and OATP1B3 (in humans) as well as the murine ortholog Oatp1b2 as transporters that regulate the initial step in the elimination of AIs. Genetic deficiency of this transport mechanism in mice was associated with elevated plasma levels of AIs and with concurrent increases in treatment-related arthralgia. In line with these findings, we found that low hepatic OATP1B-type transporter activity in patients with breast cancer was associated with an increase in AI-associated arthralgia. These findings shed light on the rate-limiting step in the elimination of AIs and suggest a rationale for the potential implementation of transporter biomarkers to predict susceptibility to AI-associated arthralgia and ultimately mitigate this debilitating toxicity.

**Significance:**

AIs are effective but often discontinued because of arthralgia. This study explores the role of OATP1B transporters in AI-related side effects and the potential usage of transporter biomarkers to predict and reduce the risk of arthralgia associated with AI treatment.

## Introduction

Aromatase inhibitors (AI), including anastrozole, letrozole, and exemestane, have long been used in the adjuvant treatment of advanced or metastatic hormone receptor–positive breast cancer in postmenopausal women (1, 2). Despite their clinical effectiveness, AIs are associated with pronounced adverse events that significantly compromise the quality of life of patients (1, 3, 4). Estrogen deprivation is currently postulated as the cause for most AI-associated side effects, which predominantly manifest as hot flashes, nausea, vomiting, and fatigue (4–6). In addition, musculoskeletal side effects, including arthralgia, joint stiffness, myalgia, and tendinopathy, have been reported in up to 74% of patients undergoing AI treatment (1, 4, 7). AI-associated arthralgia (AIAA) is characterized by symmetrical joint pain and stiffness affecting the knees, wrists, hips, shoulders, and ankles (1). The onset of arthralgia, on average, occurs 1 to 2 months after therapy initiation but can manifest at any point and leads to premature treatment discontinuation in 30% of cases, which in turn is associated with a high risk of cancer relapse (5).

Given that AIs are primarily used in the adjuvant setting, their administration may extend from 5 to 10 years, depending on an individual’s risk of relapse; however, only 50% to 68% of patients maintain full compliance with treatment beyond 3 years (1, 5). The exact mechanism behind AIAA remains elusive, but several genes have been associated with a higher risk of arthralgia development, including those involved in bone remodeling, metabolism, and transport (5, 7). In this context, it is noteworthy that AIs exhibit extensive interindividual pharmacokinetic variability, with up to 12-fold differences in plasma levels observed in patients receiving the same dose (7, 8). The mechanisms underlying the unpredictable pharmacokinetic properties of AIs remain largely unexplained, although it has been speculated that a critical determinant of the observed variability is associated with differential expression of polymorphic drug-metabolizing enzymes and/or transporters at sites of elimination (6, 7, 9). Although the metabolic pathways of AIs have been reasonably well established, the mechanisms by which AIs are taken up into human liver cells, in advance of metabolism, are unknown. However, previous studies have provided evidence that the cellular uptake of estrogens and related hormonal therapeutics used in the treatment of breast cancer, such as tamoxifen, may be regulated by the polymorphic organic anion-transporting polypeptides OATP1B1 (gene name, *SLCO1B1*) and/or OATP1B3 (gene name, *SLCO1B3*; refs. 10–12). These transporters are expressed at high levels in the liver, in which their localization is restricted to the basolateral membrane of hepatocytes, and they have been implicated in the liver uptake of multiple structurally diverse endogenous molecules and xenobiotics (13). In the current study, using *in silico*, *in vitro*, and *in vivo* approaches in mice and humans, we tested the hypothesis that OATP1B-type transporters are determinants of interindividual variability in the pharmacokinetics of AIs, and that genetic defects in this transport mechanism predispose to increased susceptibility to AIAA.

## Materials and Methods

### Chemicals and reagents

Anastrozole, letrozole, and exemestane were purchased from Sellekchem. Reference standards of letrozole, and exemestane used as an internal standard for the analytic method, were purchased from Toronto Research Chemicals Inc. Reagents for LC/MS-MS were as previously described (14). [^3^H]-estradiol-17β-D-glucuronide (EβG; specific activity, 50.1 Ci/mmol) was purchased from American Radiolabeled Chemicals. 8-(2-[Fluoresceinyl]-aminoethylthio)-adenosine-3′,5′-cyclic monophosphate (8-FcA) was obtained from Axxora, LLC. Cholecystokinin sulfate (CCK-8) was purchased from Cayman Chemical. Standard cell culturing procedures were conducted using DMEM and FBS obtained from Gibco. Poly-D-lysine for coating plates was purchased from MP Biomedicals. Pierce BCA protein assay kits were purchased from Thermo Fisher Scientific, and 8-acetoxypyrene-1,3,6-trisulfonate (ACE) was purchased from Carbosynth Limited. Cyclosporin A was purchased from TCI Chemicals.

### Cell culture

Human embryonic kidney cells (HEK293; RRID: CVCL_0045) stably transfected with human OATP1B1 (*SLCO1B1*) cDNA, Oatp1b2 (*Slco1b2*) cDNA, OATP1B3 (*SLCO1B3*) cDNA, or an empty control vector were established and cultured as previously described (15). All the cells were maintained in DMEM supplemented with 10% FBS. OATP1B1-overexpressing cells were grown in the presence of 50 μg/mL hygromycin B and 15 μg/mL blasticidin, and OATP1B3-overexpressing cells in the presence of 1 mg/mL G418. All the cell lines were maintained in a humidified incubator at 37°C and 5% CO_2_. Prior to plating, 12-well tissue culture plates (Thermo Fisher Scientific) were coated with poly-D-lysine. Seeding media were prepared using phenol red–free DMEM with 10% FBS with no selection agents. To induce expression of OATP1B1, 1 μg/µL of doxycycline was added prior to seeding the cells.

### Transport inhibition assays

Each of the OATP1B-overexpressing cells and vector control cells was plated in 12-well tissue culture plates at a volume of 1 mL (containing 4 × 10^6^ cells/mL) per well. Plates were kept in an incubator at 37°C, and transport assays were conducted 24 hours later or when the cells were confluent. Transport activity was measured using EβG, CCK-8, and 8-FcA as prototypical substrates of OATP1B. Experiments were conducted using phenol red–free and serum-free DMEM. Intracellular levels of total radioactivity originating from EβG or CCK-8 were assessed by liquid scintillation counting. The resulting radiation counts were normalized to total protein levels as determined by a Pierce protein assay. The influence of anastrozole, letrozole, or exemestane on OATP1B-mediated transport was assessed following 15 minutes of pre-incubation at various concentrations of test inhibitors followed by co-incubation of test inhibitors with EβG or CCK-8. Inhibition of transport activity by AIs was determined by comparing the accumulation of EβG or CCK-8 in the presence of anastrozole, letrozole, or exemestane to that of EβG or CCK-8 in the presence of only the control vehicle, DMSO. The final concentration of DMSO in media was less than 0.2% in all *in vitro* experiments.

Transport inhibition assays using 8-FcA were completed as previously described (16). OAT1B-overexpressing cells and vector control cells were grown to confluency in a 96-well plate. The cells were washed and then incubated for 15 minutes with 50 μL phenol red–free DMEM containing AIs or vehicle (DMSO). The media were aspirated off and the cells were co-incubated with 70 μL media containing 5 μmol/L or 3 μmol/L 8-FcA with test inhibitors (OATP1B1 and OATP1B3 cells, respectively) for 15 minutes. The media were removed, and the cells were washed three times with ice-cold PBS. Fluorescence was measured on a microplate reader at an absorbance wavelength of 485 nm and emission wavelength of 535 nm. The cells were lysed with 35 μL of cell lysis buffer at 4°C overnight and a Pierce protein assay was used to normalize the fluorescence reads to total protein.

### Competitive counterflow assays

The assay was conducted using the fluorescent probe ACE in which OATP1B-overexpressing cells or vector control cells were seeded in 96-well plates in 200 μL DMEM (containing 5 × 10^4^ cells/well) and incubated for 24 hours or until confluent. The medium was aspirated off, and the cells were washed three times with PBS at room temperature. The cells were then treated with 100 μL of 2 μmol/L or 10 μmol/L ACE (OATP1B1 or Oatp1b2/OATP1B3 cells, respectively) and incubated at 37°C for 20 minutes in the uptake buffer. The supernatant was removed, and 100 μL of above indicated ACE alone or in combination with DMSO or AIs (10–200 μmol/L) in uptake buffer were added to the cells. The plates were incubated for another 20 minutes at 37°C. The supernatant was removed, and the cells were washed three times with 200 μL of ice-cold PBS. Lastly, 200 μL of 0.1 N NaOH were added to cells and incubated for 20 minutes at room temperature. Fluorescence was measured on a microplate reader at an absorbance wavelength of 460 nm and emission wavelength of 510 nm. The intracellular concentrations of ACE were calculated by comparison of fluorescence values observed with control samples incubated with ACE alone, which were set to 100% (17).

The competitive counterflow assay was also conducted using the radiolabeled probe EβG, and employed OATP1B1- and OATP1B3-overexpressing cells seeded in 96-well plates and grown till confluency (18). These cells were preloaded to saturation at room temperature with 0.01 μmol/L EβG in pre-warmed serum- and phenol red–free media for 1 hour. Following loading, efflux of the radiolabeled probe resulted from the introduction of 1 μL stock solution of 0.1, 1, and 10 mmol/L test compound in DMSO (or glucose control). Following a 30-minute post-incubation of test compound, the transport activity was terminated by a 3× wash with 4°C PBS. Finally, the cells were solubilized with 150 μL of 1% Triton X-100. To determine final radioactivity, 100 μL of this solution were transferred to 96-well isoplates. To each sample-containing well, 200 μL of scintillation fluid were added, mixed, and total radioactivity was determined using a MicroBeta microplate scintillation counter (PerkinElmer).

### Determination of drug levels

Liquid chromatography-tandem mass spectrometry (LC/MS-MS) was used to quantify exemestane concentrations. The analysis was conducted using a Vanquish UHPLC aligned with a Quantiva triple-stage quadrupole mass spectrometer (Thermo Fisher Scientific). An ACQUITY UPLC BEH C18 Column (130 Å, 1.7 μm, 2.1 mm × 50 mm, Waters Co.) protected by ACQUITY UPLC BEH Shield RP18 VanGuard Pre-column (130 Å, 1.7 μm, 2.1 mm × 5 mm, Waters Co.) was used for analyte separation. The injection volume of the sample was 10 μL. The temperature of the column was kept at 40°C, whereas the autosampler rack was kept at 4°C. The mobile phase consisted of solvent A, which was a solution of 0.1% acetic acid in LC/MS-grade water, and solvent B, which was a solution of 0.1% formic acid in acetonitrile. The total run time was 4.5 minutes. The following were the gradient conditions: 0.3 mL/minutes was the flow rate for the following intervals: 0 to 0.5 minutes, 30% B; 0.5 to 3.0 minutes, 30% to 95% B; 3.0 to 4.0 minutes, 95% B; and 4.001 to 4.5 minutes, 95% to 30% B. The vaporizer was adjusted to 360°C, and the ion transfer tube temperature was 340°C. The mass spectrometry assay was configured with a positive voltage of 3600 V delivered to the ESI capillary. The collision gas utilized was argon, with a pressure of 1.5 mTorr. The mass spectrometer was set up with the following optimized parameters: 1 arbitrary unit (Arb) for sweep gas, 5 Arb for auxiliary gas, and 25 Arb for sheath gas. Precursor molecular ions and product ions were recorded for confirmation and detection of exemestane (297→121) and using [^2^H_3_]-exemestane-13C as an internal standard (300→121). The accuracy, expressed as a percent bias varied from −7.80% to 5.10%, whereas the inter-assay and intra-assay precisions ranged from 2.19% to 7.09% according to the results of assay validation tests. The lower limit of quantification was 0.4 ng/mL.

Letrozole was quantified using an LC/MS-MS method described previously (14). A Vanquish UHPLC and Quantiva triple-stage quadrupole mass spectrometer (Thermo Fisher Scientific) were used in the analysis. Analytes were separated chromatographically using a C18 AQUASIL guard cartridge (2.1 mm × 10 mm, 3 μm; Thermo Fisher Scientific) and an Accucore aQ column (50 mm × 2.1 mm, 2.6 μm). The temperature of the column was set at 40°C, whereas the autosampler was kept at 4°C. The mobile phase consisted of solvent A, which was a solution of 0.1% acetic acid in LC/MS-grade water, and solvent B, which was a solution of 0.1% formic acid in acetonitrile. A gradient elution method was employed for 5.0 minutes, with a flow rate of 0.4 mL/minutes. The gradient conditions were as follows: a 5% concentration of B was used for the first 0.5 minutes, which was gradually increased to 95% at 4.0 minutes; between 4.0 and 4.5 minutes, the concentration of B remained constant at 95%; between 4.50 and 4.51 minutes, it was reduced from 95% to 5%; and between 4.51 and 5.0 minutes, it was kept at 5%. Injections were performed using 3 μL of the extracted samples. The mass spectrometer parameters were optimized and configured as follows: 25 Arb for sheath gas, 5 Arb for auxiliary gas, and 1 Arb for sweep gas. The temperature of the ion transfer tube and vaporizer was adjusted to 340°C and 360°C, respectively. Argon was used as the collision gas at a pressure of 1.5 mTorr. Letrozole was confirmed and detected by recording precursor molecular ions and product ions (286.1→217.0), with [^2^H_4_]-letrozole serving as an internal standard (290.0→221.0). Results from assay validation studies revealed that the within-day precision and between-day precision ranged from 3.43% to 13.0%, and the accuracy ranged from −0.66% to 6.40%. The lower limit of quantification was 5 ng/mL.

### Animal studies

In consideration of the use of AIs in the treatment of breast cancer, a disease most frequently found in women, only female mice were used in the present *in vivo* studies. Because the pharmacokinetic profile of AIs such as letrozole is sexually dimorphic in rodents but not in humans (19), and since hepatic expression levels of Oatp1b2 are not dependent on sex (20), the reported *in vivo* data are expected to be relevant for both sexes.

### *In vivo* pharmacokinetic studies

Female wild-type, Oatp1a/1b cluster-knockout mice [Oatp1a/1b^(−/−)^ mice] and Oatp1a/1b^(−/−)^ mice with transgenic hepatic expression of OATP1B1 [OATP1B1^(tg)^ mice] or OATP1B3 [OATP1B3^(tg)^ mice] were all on an FVB background (RRID: IMSR_T000141), and experimental groups were age- and weight-matched for each experiment. Female mice with a deficiency of all Cyp3a isoforms [Cyp3a^(−/−)^] were similarly matched with FVB wild-type mice. All mice were bred in-house. Littermates of the same sex and genotype were housed in groups of maximally five animals in a temperature- and light-controlled environment (12-hour light/dark cycles) in cages lined with absorbent bedding. Mice were given free access to standard food and water. All breeding and experimental procedures were conducted with the approval of the Institutional Animal Care and Use Committee at The Ohio State University under protocol number 201500000101-R2 approved on 9/20/2021.

For *in vivo* pharmacokinetic studies, mice were randomized into experimental groups of equal size. Dosing solutions of letrozole and exemestane were prepared by dissolving the respective drug in DMSO (10%) and then adding polyethylene glycol 300 (40%), polysorbate 80 (Tween-80; 5%), and saline (45%) stepwise to create a 2 mg/mL suspension. Letrozole and exemestane were administrated orally at doses of 10 mg/kg and 20 mg/kg, respectively. Pharmacokinetic studies were performed as described previously (21). After a single dose of AI, whole-blood samples of approximately 30 μL were collected from each mouse at six different time points. Samples collected at the first three time points were collected from the submaxillary vein using a sterile, disposable 4- or 5-mm Goldenrod animal blood lancet and collected into a glass micro-hematocrit heparinized capillary tube. For samples at the fourth and fifth time points, mice were anesthetized using 2% isoflurane and whole blood was obtained from the retro-orbital venous plexus using capillary tubes. The final sample was obtained by cardiac puncture after euthanizing the mice via carbon dioxide inhalation. All samples were centrifuged at 13,000 rpm for 5 minutes, and the plasma supernatant was immediately transferred to a 0.5-mL tube on dry ice and stored at −80°C until analysis.

Pharmacokinetic parameters were obtained using noncompartmental analyses with Phoenix WinNonlin version 8.1 (Certara). The peak plasma concentration and the area under the plasma concentration–time curve (AUC) were used as pertinent measures of systemic exposure.

### Molecular docking

To evaluate AIs as potential substrates of OATP1B1 and OATP1B3, molecular *in silico* docking of AIs in recently reported cryogenic electron microscopy structures of OATP1B1 and OATP1B3 was performed. For OATP1B1, several conformational states have been reported by Shan and colleagues (22) and Ciută and colleagues (Ref. 23; PDB codes: 8HNB, 8HNC, 8HNH, 8K6L, 8HND, and 8PHW) in both apo and substrate-bound forms (Supplementary Table S1). Docking simulations were conducted for each conformation state, and the poses with the best docking scores were selected for visualization (Supplementary Fig. S1A). Protein structures were prepared and minimized using the OPLS3e force field in the Protein Preparation Wizard of Schrödinger 2020.1 (LLC, 2020). The ligand diameter midpoint was set to 30 Å, and drug conformations were prepared using the LigPrep Wizard. Docking was performed using the Glide SP module.

In the case of OATP1B3, only one conformational state has been reported (PDB code: 8PG0), in which it is in complex with bicarbonate (23). However, the binding site of bicarbonate is suggested to be an allosteric site that might induce conformational changes in OATP1B3, preventing it from binding drug substrates. As the overall sequence identity between OATP1B3 and OATP1B1 is 75%, two homology models of OATP1B3 were constructed using two OATP1B1 conformational states (8HNC and 8PHW) as templates. The substrate-binding site of OATP1B3 was assigned to the same region as that of OATP1B1. Template 8HNC adopts a minor pocket–closed conformational state, whereas template 8PHW adopts a minor open state. After homology modeling using SwissModel (https://swissmodel.expasy.org/), OATP1B3-Model1 adopts a minor pocket–closed conformational state (Supplementary Fig. S1B). However, OATP1B3-Model2, based on the 8PHW template, exhibits a blocked minor pocket because of the side chain of F352, despite the template 8PHW being in a minor pocket–open state. To address this, we manually adjusted the side chain of F352 to the minor pocket–open position, resulting in Adjusted-OATP1B3-Model2 (Supplementary Fig. S1C), followed by protein minimization using Schrödinger. Subsequent steps were consistent with those employed for OATP1B1. The grid for each PDB was generated by centering on the original ligand, except for the apo form (8HNB), which centered on three conservative hydrophobic residues in the substrate-binding sites (Supplementary Fig. S1D).

### Von Frey hair test

Von Frey filaments were used as a mechanical sensitivity measurement following administration of letrozole in both chronic and acute treatments, using a strategy developed previously (24). After ensuring that the animal is acquainted with the environment, joint pain was directly evaluated by a hind limb withdrawal response to mechanical stimulation applied by the filaments. We leveraged this technique to evaluate the joint pain in wild-type mice, Oatp1a/1b^(−/−)^ mice, and humanized transgenic mice treated with letrozole doses ranging from 0.1 to 0.5 mg/kg. After a single dose, mechanical sensitivity was tested with the Von Frey filaments at 0, 1, 3, 6, and 24 hours after drug administration (25, 26). During the chronic treatment of letrozole (0.1 mg/kg/day for 15 days), mechanical sensitivity was measured at baseline, at the end of week 1, and the end of week 2. Analysts were blinded to the composition of the treatment groups.

### Photobeam activity open field system

An open-field photobeam activity system was used to monitor the motor activity of mice over a period of 12 hours after administration of letrozole (27, 28). Spontaneous physical/locomotor activity was assessed using an activity monitoring system consisting of a two-dimensional (X and Y axes) square frame that emits a grid of infrared beams (IR Actimeter and Actitrack software, Panlab, Harvard Apparatus). Animals were placed in a transparent enclosure inside the frame, and movement within the enclosure (general locomotor activity) was recorded as interruptions in the beams. During acute studies, mice were dosed with letrozole (0.5 mg/kg) orally at 5:00 pm and were tested individually for 12 hours during the active/dark phase of the light cycle (6:00 pm–6:00 am). Physical activity of the mice was similarly monitored at baseline and at the end of 15 days of the chronic treatment.

### Histopathology

At the end of the chronic letrozole treatment, hind limbs of three randomly selected mice per group were collected, peeled for skin, and preserved in 10% neutral buffered formaline at room temperature for at least 48 hours until tissue processing. Tissues from the hind limbs were routinely processed for histopathology on a Leica Peloris 3 Tissue Processor (Leica Biosystems), embedded in paraffin, sectioned at an approximate thickness of 4 to 5 μm, and batch stained with hematoxylin and eosin on a Leica ST5020 autostainer using a routine and quality-controlled protocol. Slides were evaluated and representative photomicrographs were taken by a board-certified veterinary pathologist using a Nikon Eclipse Ci-L Upright Microscope (Nikon Instruments, Inc.) with attached 18 megapixel Olympus SC180 microscope-mounted digital camera and cellSens imaging software (Olympus Life Science).

### Human samples

The experimental protocol was approved by The Ohio State University Human Institutional Review Board (number 2007C0066). Studies were conducted according to Declaration of Helsinki principles, and all subjects provided written informed consent (29). Patients were randomly selected from previous studies in which they were categorized into two groups comprising either asymptomatic patients and those with clinically diagnosed AIAA (defined as grade 2 or above by NCI Common Terminology Criteria for Adverse Events v4) and/or requiring amendment or discontinuation of AI therapy (29). Patients did not concurrently receive other chemotherapeutic agents that have a black box warning for potential to cause arthralgia, and patients did not receive other medications that are known to influence the function of OATP1B-type transporters.

### Statistical analysis

Data are presented as mean ± SD, unless stated otherwise. Group differences were assessed for statistical significance using an unpaired Student *t* test (two groups) or two-way ANOVA (>2 groups) for normally distributed data, whereas the Mann–Whitney–Wilcoxon rank-sum test (U test, two groups) or Kruskal–Wallis test (>2 groups) was used to analyze data deviating from the normal distribution. For multiple comparisons, Dunnett multiple comparison adjustment or Tukey multiple comparison adjustment was applied to normally distributed data where appropriate, and a Bonferroni multiple comparison adjustment was applied to data deviating from the normal distribution if needed. To evaluate the difference in chenodeoxycholate-24-glucuronide (CDCA-24G) baseline plasma concentrations between patients with and without arthralgia symptoms, we anticipated a within-group difference of 20% and a between-group difference of 40% based on our previous studies (30). As such, eight patients per group (with 10% attrition, nine patients) would achieve a power of 96.0% at a significance level of α = 0.05. All statistical tests were two-tailed, and *P* < 0.05 was considered statistically significant across all the studies. Statistical tests were conducted using GraphPad Prism 10.0.2 (GraphPad Software, RRID: SCR_002798).

### Data availability

Raw data are available upon reasonable request from the corresponding author.

## Results

### *In silico* docking of AIs into OATP1B-type transporter models

**Figure 1:**
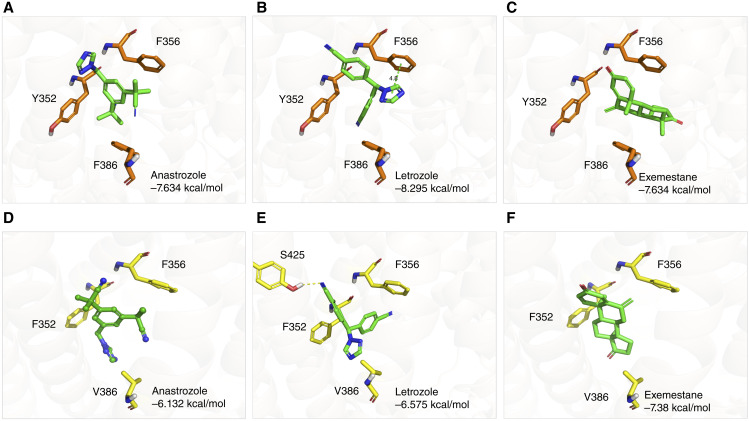
Docking poses of AIs to OATP1B1 and OATP1B3. Best docking poses of AIs to (**A–C**) OATP1B1 and (**D–F**) OATP1B3. Orange, OATP1B1 residues; green, docking poses of selected drugs; green dashed line, pi–pi interactions; yellow, OATP1B3 residues; and yellow dashed line, hydrogen bonds.

We initially hypothesized that the AIs anastrozole, letrozole, and exemestane could be transported substrates of OATP1B-type transporters based on existing evidence for structurally related endogenous hormones and xenobiotics (13, 31). Preliminary examination of a potential interaction of AIs with these transporters was performed using computational analyses employing recently reported cryogenic electron microscopy structures of OATP1B1 and OATP1B3 (22, 23). The docking outcomes for the selected drugs with OATP1B1 (Supplementary Table S2) and the corresponding docking poses with the best scores (Fig. 1A–C) revealed that all three drugs exhibited favorable interactions with the minor pocket–open states 8HND or 8PHW (22). Notably, there were no observed polar contacts between these drugs and OATP1B1, suggesting that their binding may be predominantly driven by hydrophobic interactions. A pi–pi interaction was identified between letrozole and F356. Furthermore, the docking poses of these drugs spatially overlap with a region occupied by estrone-3-sulfate, a known endogenous substrate of OATP1B1, further supporting the notion that AIs may undergo OATP1B1-mediated transport (Supplementary Fig. S2A–S2C).

Similarly, the docking results for OATP1B3 homology models (Supplementary Table S3) indicated improved docking scores for the three AIs in Adjusted-OATP1B3-Model2 (minor pocket–open) compared with OATP1B3-Model1 (minor pocket–closed). This finding suggests a similar preference for a minor pocket–open binding mode in OATP1B3-mediated transport compared with OATP1B1. However, the orientation of these drugs differs between OATP1B1 and OATP1B3, potentially influenced by the shape difference of the minor pocket. The phenylalanine at residue 386 in OATP1B1 is bulkier than the valine at the equivalent position in OATP1B3, resulting in a narrower minor pocket in OATP1B1 (Fig. 1A–C vs. Fig. 1D–F). Consequently, the triazole fragments of anastrozole and letrozole are allowed to orient toward V386 in OATP1B3, a configuration that may be less favorable in OATP1B1 due to space limitation, whereas for letrozole, an additional hydrogen bond with S425 may contribute to the interaction with OATP1B3.

### *In vitro* interaction of AIs with OATP1B-type transporters

**Figure 2:**
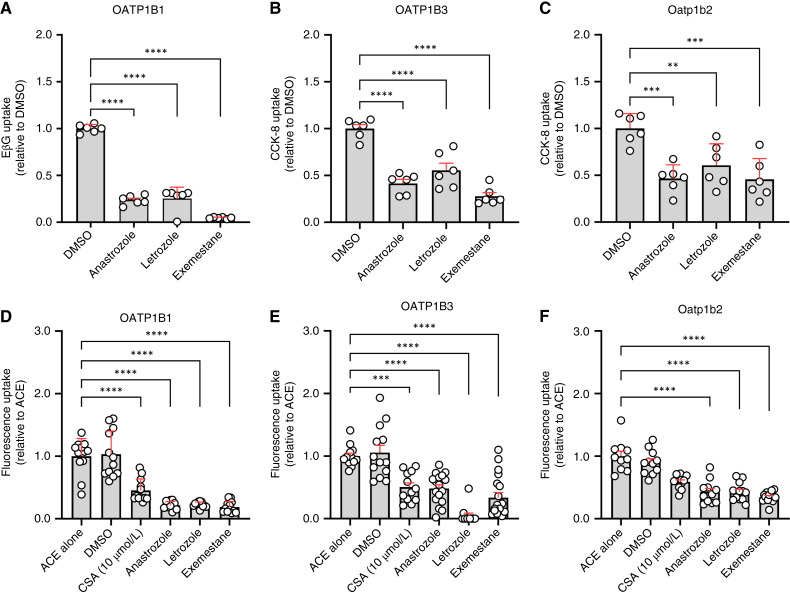
AIs are inhibitors and substrates of OATP1B transporters. **A,** Inhibition of EβG (0.2 μmol/L) uptake in the OATP1B1-overexpressing HEK293 cells in the presence of AIs (100 μmol/L). AIs (100 μmol/L) inhibit CCK-8 uptake in the OATP1B3-overexpressing (**B**) and Oatp1b2-overexpressing (**C**) HEK293 cells. Transporter-mediated uptake of AIs (100 μmol/L) in OATP1B1- (**D),** OATP1B3- (**E**), and Oatp1b2-overexpressing (**F**) HEK293 cells was assessed by competitive counter flow assay. Cellular uptake was measured indirectly via the steady state reduction of ACE (2 μmol/L or 10 μmol/L ACE in OATP1B1 or OATP1B3/Oatp1b2 cells, respectively) fluorescent signal compared with the ACE alone group. CSA, cyclosporin A. Data are shown as mean ± SD. One-way ANOVA with Dunnett multiple comparison adjustment was used to analyze the data. **, *P* < 0.01; ***, *P* < 0.001; ****, *P* < 0.0001 denotes compared with DMSO or ACE groups (*n* = 6–12 per group).

To verify the *in silico* docking analyses, we next examined the *in vitro* interaction of AIs with OATP1B-type transporters using genetically engineered HEK293 cells overexpressing human OATP1B1, human OATP1B3, or the single murine ortholog, Oatp1b2. We initially focused on evaluating the inhibitory properties of AIs on the transporters based on the expectation that transported substrates would act as inhibitors and impede the cellular uptake of known substrates. Indeed, these studies indicated that pre-incubation of cells with AIs inhibited the OATP1B1-, OATP1B3-, or Oatp1b2-mediated transport of several known substrates, including the estrogen conjugate EβG, the cholecystokinin derivative CCK-8, and 8-FcA (Fig. 2A–C; Supplementary Fig. S3A–S3C). The observed inhibitory properties were consistent with the ability of AIs to also affect the intracellular accumulation and retention of the fluorescent compound ACE using a competitive counterflow assay (17, 18, 32) at steady state in cells overexpressing OATP1B-type transporters (Fig. 2D–F; Supplementary Fig. S3D–S3F). Similar results were obtained when using radiolabeled EβG (Supplementary Fig. S3G–S3I), suggesting that AIs are recognized as both substrates and inhibitors of the main mammalian, liver-specific OATP1B-type transporters.

### Impact of AIs on endogenous biomarkers of Oatp1b2 function

**Figure 3:**
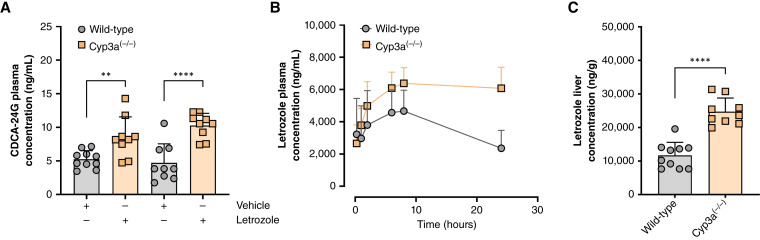
AIs inhibit OATP1B transporter activity *in vivo*. **A,** The levels of CDCA-24G biomarker in plasma were measured via LC/MS-MS in wild-type and Cyp3a^(−/−)^ mice treated with a single oral administration of letrozole (10 mg/kg) or vehicle. At 2 hours, the observed time to peak concentration of the CDCA-24G plasma concentration–time profile, the letrozole-treated group had significantly higher biomarker levels than the vehicle-treated mice. **B,** Pharmacokinetic profile and (**C**) liver concentration after a single oral administration of letrozole (10 mg/kg) treatment in wild-type and Cyp3a^(−/−)^ mice. Data are shown as mean ± SD. Two-sided, unpaired Student *t* tests were used to analyze the data and no multiple comparison adjustment was applied. **, *P* < 0.01 and ****, *P* < 0.0001 compared with the wild-type group; *n* = 9 to 10 per group. Letrozole plasma and liver concentrations were quantified using LC/MS-MS.

Previously, we identified the bile acid CDCA-24G as a selective and sensitive biomarker of hepatic Oatp1b2 function in mice and of OATP1B1/OATP1B3 function in patients with cancer (30). In advance of pharmacokinetic studies, we verified that AIs such as letrozole can cause marked inhibition of Oatp1b2 function, as measured by an increase in the circulating concentration of CDCA-24G in wild-type mice (Fig. 3A). The experiment was concurrently performed in mice lacking Cyp3a isoforms [Cyp3a^(−/−)^ mice], the enzymes responsible for the metabolism of letrozole to its carbinol metabolite (33). In wild-type mice and Cyp3a^(−/−)^ mice, concentrations of CDCA-24G in plasma were increased 1.62-fold and 2.17-fold, respectively, in animals receiving letrozole as compared with animals receiving the vehicle control (Fig. 3A). The larger inhibitory effect on hepatic Oatp1b2 function in Cyp3a^(−/−)^ mice is consistent with the observation that plasma and hepatic levels of letrozole itself were elevated in these animals compared with the levels observed in wild-type mice (Fig. 3B and C; Table 1).

**Table 1:** Pharmacokinetic parameters of letrozole in mice

Mouse genotype	Treatment (dose mg/kg)	C_max_ (ng/mL)	AUC_0–24h_ (ng × hour/mL)	AUC fold change vs. WT
Wild-type	Letrozole (10)	5,796 (±443)	88,668 (±6,642)	
Cyp3a^(−/−)^	Letrozole (10)	6,820 (±342)*	141,215 (±7,049)*	1.59 (1.34–1.90)

Each row represents 8 to 10 female mice receiving letrozole (10 mg/kg). Values represent the mean ± SEM.Two-sided Student *t* tests were used to assess the difference between wild type and Cyp3a^(−/−)^. * denotes *P* < 0.05 versus wild-type (*n* = 8–10 per genotype). The 95% confidence interval is listed for the AUC ratio between Cyp3a^(−/−)^ genotype and wild-type groups.

Abbreviations: AUC_0–24h_, area under the concentration–time curve from time 0 to 24 hours; C_max_, maximum plasma concentration; WT, wild type.

### Transport of AIs by OATP1B-type transporters *in vivo*

**Figure 4:**
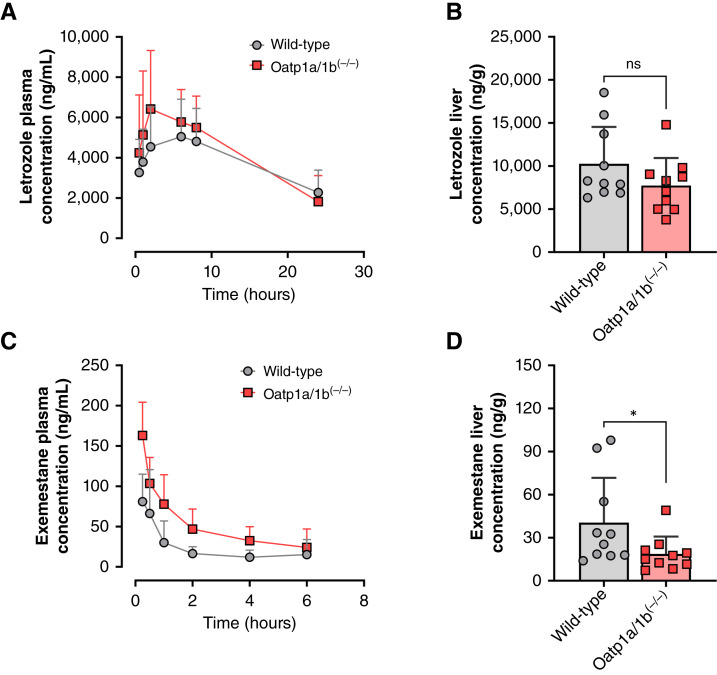
OATP1B deficiency results in elevated AI plasma exposure and decreased liver accumulation. **A,** Pharmacokinetic profile of letrozole after a single oral dose of 10 mg/kg in wild-type and Oatp1a/1b^(−/−)^ mice. **B,** Liver letrozole concentration of represented genotypes. **C,** Pharmacokinetic profile of exemestane after a single oral dose of 20 mg/kg in wild-type and Oatp1a/1b^(−/−)^ mice. **D,** Liver exemestane concentration in the representative genotypes. Plasma concentrations of letrozole and exemestane were analyzed by LC/MS-MS. The maximum plasma concentration C_max_ and AUC_0–6h_ values are summarized in Table 1. Pharmacokinetic parameters (*n* = 8–10 per genotype) were calculated by noncompartmental analysis using Phoenix WinNonlin 8.1. Data are shown as mean ± SD. Two-sided, unpaired Mann–Whitney–Wilcoxon rank tests were used to analyze the data and no multiple comparison adjustment was applied. NS, non-significant; *P* = 0.279 and *, *P* < 0.05 compared with the wild-type group.

To evaluate the *in vivo* interaction of AIs and OATP1B-type transporters, we performed pharmacokinetic studies in wild-type mice and Oatp1b2-deficient mice that also lacked all murine Oatp1a-type transporters [Oatp1a/1b^(−/−)^ mice]. Consistent with the notion that AIs serve as substrates of OATP1B-type transporters, we found that Oatp1a/1b^(−/−)^ mice exhibited elevated plasma concentrations of letrozole and exemestane compared with wild-type mice, an observation that was also accompanied by higher levels of both letrozole and exemestane in livers of wild-type mice (Fig. 4A–D; Table 2). Although Oatp1a/1b^(−/−)^ mice with transgenic overexpression of human OATP1B1 or human OATP1B3 in the liver (34–36) did not recapitulate the pharmacokinetic profile observed in wild-type mice, liver concentrations of letrozole and exemestane were partially restored, suggesting that OATP1B-type transporters are responsible, at least in part, for the uptake of AIs in hepatocytes (Table 2; Supplementary Fig. S4A–S4D).

**Table 2:** Influence of the OATP1B genotype on AI pharmacokinetics

Mouse genotype	Treatment (dose mg/kg)	C_max_ (ng/mL)	AUC_0–6h_ (ng × hour/mL)	AUC fold change vs. WT
Wild-type	Letrozole (10)	5,180 (±660.0)	25,466 (±3,311)	
OATP1B1^(tg)^	Letrozole (10)	6,547 (±769.0)	33,030 (±3,335)	1.29 (0.94–1.78)
OATP1B3^(tg)^	Letrozole (10)	6,674 (±1,058)	32,426 (±5,667)	1.27 (0.83–1.95)
Oatp1a/1b^(−/−)^	Letrozole (10)	6,747 (±819.0)	33,255 (±4,104)	1.3 (0.92–1.86)
Wild-type	Exemestane (20)	94.0 (±16)	130 (±18)	
OATP1B3^(tg)^	Exemestane (20)	152 (±26)*	294 (±48)*	2.27 (1.49–3.44)
Oatp1a/1b^(−/−)^	Exemestane (20)	159 (±13)*	292 (±32)*	2.25 (1.60–3.18)

Each row represents 8 to 10 female mice receiving letrozole (10 mg/kg) or exemestane (20 mg/kg). Values are the mean with SEM. Two-sided Student *t* tests were used to assess the group-wise differences between wild-type and transgenic animals without multiple comparison adjustment. *P < 0.05 compared with wild-type, respectively (*n* = 8–10 per genotype). The 95% confidence interval is listed for the AUC ratio between OATP1B genotype and wild-type groups.

Abbreviations: AUC_0–6h_, area under the concentration–time curve from time 0 to 6 hours; C_max_, maximum plasma concentration; WT, wild type.

### Influence of Oatp1b2 deficiency on AIAA

**Figure 5:**
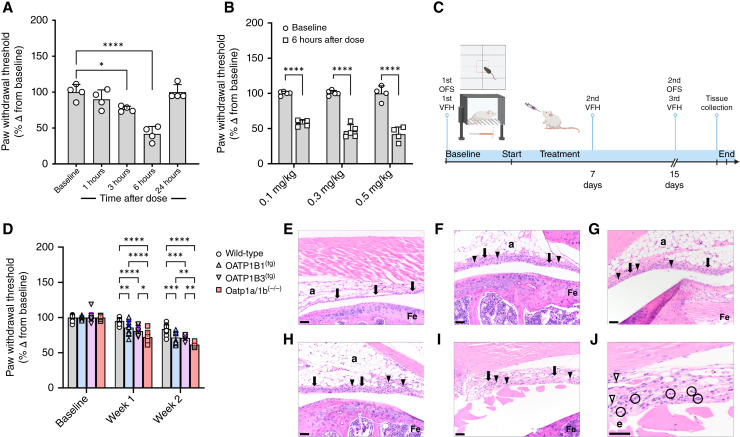
OATP1B genotype–dependent letrozole-induced arthralgia. **A,** Paw withdrawal threshold, expressed as a percentage change in paw withdrawal force (grams) from baseline, in wild-type mice at baseline and 1, 3, 6, and 24 hours after administration of a single dose of letrozole (0.5 mg/kg; *n* = 4). **B,** Paw withdrawal threshold was assessed after 6 hours in wild-type mice following administration of 0.1, 0.3, and 0.5 mg/kg of letrozole (*n* = 4–6). **C,** Letrozole chronic treatment study scheme. Mice baseline Von Frey hair test (VFH) and physical activity [open field system (OFS)] were recorded followed by daily administration of letrozole (0.1 mg/kg) for 15 days. Mechanical sensitivity was again assessed at the end of week 1 and day 15 and the physical activity after the last VFH. At the end of the experiment, mice hind legs were collected for the knee histopathology analysis. **D,** Mechanical sensitivity changes from baseline were monitored in wild-type, Oatp1a/1b^(−/−)^ mice with transgenic hepatic expression of OATP1B1 (OATP1B1^(tg)^ mice) or OATP1B3 (OATP1B3^(tg)^ mice) and Oatp1a/1b^(−/−)^ mice after 1 and 2 weeks of daily letrozole (0.1 mg/kg) administration (*n* = 10 per group). Hematoxylin and eosin staining of femurs isolated from (**E**) wild-type control, (**F**) wild-type letrozole, (**G**) OATP1B1^(tg)^ letrozole, (**H**) OATP1B3^(tg)^ letrozole, and (**I** and **J**) Oatp1a/1b^(−/−)^ letrozole–treated representative mice. a, adipose tissue; Fe, femur; arrow indicates synovium; arrowheads indicate inflammatory cells; e, edema; circles indicate close up neutrophils; and open arrow heads indicate activated vascular endothelium. Scale bar, 50 μm (**E–I**); 115 μm (**J**). Note the increase in layers and thickness of the synovial epithelium in the treated groups except for the Oatp1a/1b^(−/−)^ example (**I** and **J**). In this section, the synovial epithelium is lost but there is edema, inflammation, and activation of vascular endothelium. In **A**, One-way ANOVA with Dunnett multiple comparison adjustment was used to analyze the day; in **B**, group-wise comparisons were assessed using two-sided, unpaired Student *t* tests without multiple comparison adjustment; in **D**, group-wise comparisons were assessed using two-way ANOVA with Tukey multiple comparison adjustment. *, *P* < 0.05; **, *P* < 0.01; ***, *P* < 0.001 and ****, *P* < 0.0001. Data are shown as mean ± SD. [**C** and **D,** Created in BioRender. Buelow, D. (2025) https://BioRender.com/o88g911.]

We next assessed the role of OATP1B-type transporters in the development of AIAA using letrozole as a test compound. This was based on the expectation that increased plasma levels in the context of Oatp1b2 deficiency would increase susceptibility to letrozole-induced toxicity. Using a Von Frey hair test to measure mechanical allodynia in response to AI treatment (28, 37), we found in preliminary studies that a single dose of letrozole already induced a reversible, concentration- and time-dependent allodynia in wild-type mice (Fig. 5A). The maximal mechanical sensitivity was observed at 6 hours after drug administration, a time point that aligns with the observed peak concentrations of letrozole in plasma after a single oral administration (Figs. 4A and 5B).

Next, we monitored the general physical activity of mice following a single letrozole dose using an open-field photobeam activity system, which is commonly used to assess knee joint pain in rodent models of osteoarthritis (27, 28, 37). In this model, Oatp1a/1b^(−/−)^ mice exhibited less physical activity and more resting time compared with wild-type mice, whereas Oatp1a/1b^(−/−)^ mice with transgenic hepatic expression of human OATP1B1 or human OATP1B3 displayed a partially rescued phenotype (Table 3). Importantly, mice receiving only the letrozole vehicle did not develop mechanical allodynia (Supplementary Fig. S5) and remained more physically active than animals treated with letrozole (Supplementary Table S4).

**Table 3:** OATP1B genotype–dependent changes in letrozole-induced changes in physical activity

Mouse genotype	Treatment (dose mg/kg)	Activ.	Stere.	Locom.	Dist. (minutes)	Res.T. (hours)	RT (%)	MS (%)	MF (%)
Wild-type	Single (0.5)	41,163	3,902	37,261	512	9.06	75.55	15.53	8.90
OATP1B1^(tg)^	Single (0.5)	31,678	2,713	28,964	377	9.88	82.34	11.08	6.60
OATP1B3^(tg)^	Single (0.5)	27,095	2,647	24,448	340	9.98	83.22	11.06	5.72
Oatp1a/1b^(−/−)^	Single (0.5)	26,674	2,180*	24,494	286	10.2	84.96	10.40	4.64
Wild-type	Chronic (0.1)	30,580	3,052	27,528	383	9.64	80.34	13.30	6.32
OATP1B1^(tg)^	Chronic (0.1)	28,800	2,892	25,909	380	9.86	82.19	11.58	6.22
OATP1B3^(tg)^	Chronic (0.1)	29,841	2,804	27,036	373	9.80	81.71	12.01	6.28
Oatp1a/1b^(−/−)^	Chronic (0.1)	26,637	2,572	24,065	316*	10.0*	83.55*	11.23*	5.23

General activity of the mice following the representative letrozole dosing regimen is summarized in the table (*n* = 5–10 per genotype).

Movement thresholds are as follows: <2.0 cm/second = resting; >2.0 cm/s and <5.0 cm/s = slow movement; and >5.0 cm/s = fast movement. Data were analyzed using two-sided Student *t* tests in comparison with representative wild-type without multiple comparison adjustment. *, *P* < 0.05 compared with the wild-type group.

Abbreviations: Activ., global activity or the sum of the stereotypes and locomotion activities; Dist, distance is the total distance (m) traveled; Locom., is conversely movement with change in position/location; MF (%), moving fast (%) is moving fast time expressed as a % of total duration (12 hours); MS (%), moving slow (%) is moving slow time expressed as a % of total duration (12 hours); Res.T, resting time is the time period (hours) during which speed is below resting threshold; RT (%), resting time (%) is resting time expressed as a % of total duration (12 hours); Stere., Stereotypes is essentially movement without change in position/location. This might include grooming, turning in place, eating, and drinking.

The influence of OATP1B-type transporters on letrozole-associated arthralgia with the drug administered as a single oral dose was also observed in chronic models in which letrozole was given daily for 15 days (Fig. 5C). In particular, Oatp1a/1b^(−/−)^ mice experienced the worst arthralgia-related phenotypic changes in mechanical sensitivity, followed by the humanized transgenic mice, and then wild-type mice (Fig. 5D). Similar genotype-dependent observations were made for letrozole-associated arthralgia based on the photobeam data that recorded physical activity (Table 3). Histopathologic examination of knee joints in mice receiving multiple doses of letrozole revealed three significant lesions consistent with synovial hyperplasia, synovitis, and patellar tendinitis. These findings were compared with a control group of treatment-naïve wild-type mice, which exhibited normal synovium (∼three epithelial cell thick). Although synovial hyperplasia was observed in all mice treated with letrozole, the most severe cases were noted in Oatp1a/1b^(−/−)^ mice (Fig. 5E–J). Notably, proliferation of small-caliber blood vessels was observed exclusively in the synovial tissues of Oatp1a/1b^(−/−)^ mice (Fig. 5I and J). Furthermore, Oatp1a/1b^(−/−)^ mice exhibited marked synovial edema (Fig. 5J) and patellar tendinitis (Supplementary Fig. S6A–S6E).

### Effect of OATP1B-type transport function on AIAA

**Figure 6:**
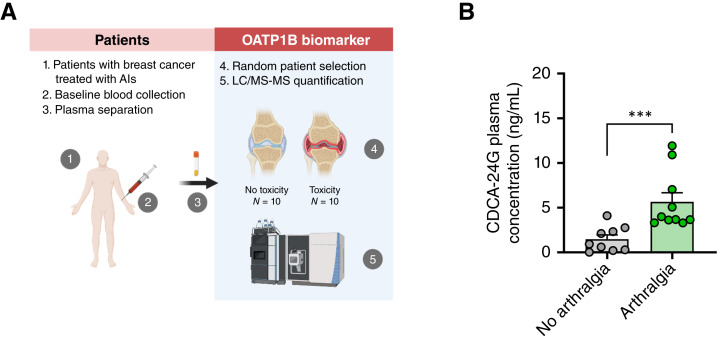
CDCA-24G baseline plasma level is significantly associated with a higher risk of AIAA development. **A,** Schematic summary of patients’ sample collection and measurement. **B,** CDCA-24G in 20 patients with breast cancer. Data were analyzed using a two-sided Mann–Whitney–Wilcoxon rank test. Data are presented as mean ± SD; ***, *P* < 0.001. [**A**, Created in BioRender. Buelow, D. (2025) https://BioRender.com/y06t832.]

To gain preliminary insights into the potential translational relevance of our *in vitro* and *in vivo* studies, we measured levels of CDCA-24G as a biomarker readout of hepatic OATP1B-type transporter function in pretreatment plasma samples of patients with breast cancer who went on to receive treatment with AIs and who either did or did not exhibit arthralgia symptoms (Fig. 6A; Supplementary Table S5). Consistent with our hypothesis that impaired OATP1B-type transporter function predisposes to AIAA, we found that pretreatment levels of CDCA-24G were significantly higher in the subset of patients that experienced this side effect during the course of therapy (Fig. 6B).

## Discussion

**Figure 7:**
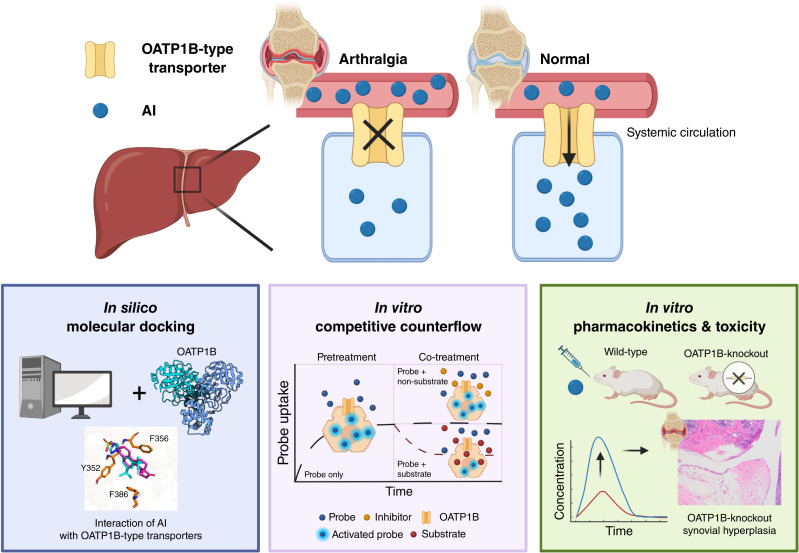
Graphical summary of evaluation of the interaction of AIs with OATP1B-type transporters. [Created in BioRender. Buelow, D. (2025) https://BioRender.com/g70l803.]

In the current study, we evaluated the interaction between AIs and OATP1B-type transporters through a comprehensive approach encompassing *in silico*, *in vitro*, *in vivo*, and clinical investigations (Fig. 7). Our findings shed light on the potential contribution of these uptake transporters to the hepatic elimination of AIs and on mechanistic underpinnings of AI-related side effects resulting from this mechanism, particularly AIAA. With emerging availability of predictive biomarkers for hepatic OATP1B-type transporter function (38), our studies offer future potential opportunities to develop strategies for personalized dosing regimens and the management of drug–drug interaction liabilities in patients with cancer requiring treatment with AIs.

The incentive for this investigation originated from the notion that AIs share structural similarities with several endogenous compounds that are known substrates of OATP1B-type transporters. Indeed, *in silico* docking analyses revealed favorable interactions between AIs and the minor pocket–open states of both OATP1B1 and OATP1B3, the main organic anion-transporting polypeptides in the human liver (39), and support the thesis that these agents are directly interacting with OATP1B-type transporters. Despite differences in orientation and a single hydrogen bond when docked to OATP1B1 and OATP1B3, the principal hydrophobic interactions of the tested AIs with these transporters remain consistent, in line with previous observations (22). The collective docking results indicate that anastrozole, letrozole, and exemestane are potential substrates of OATP1B1 and OATP1B3, exhibiting binding patterns that resemble previously reported substrate-binding modes. Ensuing studies verified this hypothesis by demonstrating that AIs in a concentration-dependent manner inhibited the uptake of various known substrates of OATP1B-type transporters, a common feature of agents that are themselves transported substrates. Furthermore, data obtained from competitive counter flow assays, an indirect method that demonstrated the utility to assess substrate affinity (32), suggested AIs as transported substrates of all human and murine OATP1B-type transporters. Although a more reliable direct cellular uptake assay overcoming the nonspecific extracellular membrane binding is warranted in the future, this finding corroborates previously reported data from retrospective studies performed in women with breast cancer receiving treatment with exemestane (8) or letrozole (10) for whom functional OATP1B1 variants were associated with altered estrogen levels and circulating AI concentrations. These findings support the thesis that uptake carriers capable of transporting AIs need to be expressed in the liver such that the drug can be taken up in advance of hepatic metabolism and thereby drive levels of AIs in the systemic circulation.

To validate the *in silico* and *in vitro* findings, *in vivo* studies were conducted in translationally relevant mouse models with variable OATP1B-type transporter function (40). Although the rodent and human OATP1B-type transporters share a high degree of sequence homology, similarity in basolateral membrane localization, and have largely overlapping substrate specificity (41), it is noteworthy that, unlike in humans, mouse hepatocytes express multiple members of Oatp1a-type transporters, a related subfamily of solute carriers that can potentially provide compensatory restoration of function when Oatp1b2 is lost (42). For this reason, *in vivo* studies were performed with letrozole and exemestane, as respective representatives of nonsteroidal and steroidal AIs, in Oatp1a/1b^(−/−)^ mice, an Oatp1b2-null model that carries an additional deficiency in all members of the Oatp1a subfamily. Interestingly, although higher plasma levels of AIs were observed in Oatp1a/1b^(−/−)^ mice because of a defect in liver uptake, transgenic Oatp1a/1b^(−/−)^ mice with hepatic expression of either human OATP1B1 or human OATP1B3 failed to show complete restoration of the phenotype recorded in wild-type mice. This finding is in line with previous studies suggesting that changes in the pharmacokinetic profile of dual OATP1B1/OATP1B3 substrates, such as the RORγ agonist cintirorgon (43) or the sorafenib metabolite sorafenib-β-D-glucuronide (15), cannot be rescued in Oatp1a/1b^(−/−)^ mice overexpressing only one of these human transporters in the liver (44). It is possible that this partial restoration is due to differences in affinity of AIs for mouse Oatp1b2 relative to human OATP1B1 and OATP1B3, and/or that the functional expression of OATP1B1 or OATP1B3 in the liver of the transgenic mice is lower than that of Oatp1b2 in the parental, wild-type strain (45). Pharmacokinetic studies in humanized mice expressing both OATP1B1 and OATP1B3 are required to determine whether in such model the phenotypes observed in wild-type mice can be faithfully recapitulated (46). Regardless of the outcome of such studies, the current findings support the possibility that even partial deficiency of either OATP1B1 or OATP1B3 function, for example as a result of an inherited genetic defect (13), can lead to a buildup of AIs in the circulation and result in altered tissue distribution, and this is consistent with clinical observations made previously with exemestane (8) and letrozole (10).

In this context, it is worth noting that Oatp1a/b deficiency in mice is not associated with any pronounced compensatory alterations in expression of other xenobiotic transporters of potential relevance to AIs in the liver (15, 47). Moreover, we previously reported that the functional expression of the key cytochrome P450 enzymes and uridine 5′-diphospho-glucuronosyltransferases associated with the mammalian metabolism of AIs, including Cyp3a and Ugt1a isoforms (48), is unchanged in these transporter knockout mice (15, 47). These findings eliminate the concern that the changes observed in plasma levels of AIs in Oatp1a/1b^(−/−)^ mice, compared with wild-type animals, might have been due to compensatory alteration in or shunting of critical elimination pathways. These results collectively support the thesis that OATP1B-type transporters play a critical role in the pharmacokinetic profile of both nonsteroidal and steroidal AIs.

Importantly, our findings provide insights into the interrelationship between OATP1B-type transporter function, altered hepatic elimination, and the development of AIAA as determined using Von Frey hair tests for mechanical hypersensitivity and an open-field photobeam system to monitor general physical activity of mice receiving letrozole. As expected, the increased plasma levels of AIs in Oatp1a/1b^(−/−)^ mice were associated with increased susceptibility to AIAA, characterized by significantly decreased mechanical sensitivity thresholds and physical activity in transporter-deficient models compared with wild-type mice, and these phenotypic alterations were observed with both acute and chronic drug dosing regimens. The observed magnitude of reduced physical activity in the context of OATP1B-type transporter deficiency is consistent with the notion that the photobeam system measurements were performed at a time when most of the administered letrozole had already been eliminated. This finding aligns with the data in the prescribing information for letrozole indicating that the agent does not substantially accumulate in the plasma of patients receiving continuous dosing regimens (49).

Histopathologic examination of knee tissue and surrounding regions following a multi-dose regimen with letrozole revealed more severe symptoms in Oatp1a/1b^(−/−)^ mice compared with wild-type mice, including synovial hyperplasia, patellar tendinitis, increased synovial membrane cellularity, and synovial thickening. Collectively, these histopathologic findings highlight the significant and varied effects of chronic letrozole treatment on the knee joints in mice, with the most severe lesions observed in Oatp1a/1b^(−/−)^ mice. The presence of neutrophils in certain cases indicated an acute inflammatory response within these tissues, shedding light on the impact of the treatments on the knee joint health of mice receiving AIs.

The potential translational significance of our observations made in mice was supported by the confirmation of an association between baseline plasma levels of CDCA-24G, a known endogenous biomarker of OATP1B-type transporter function, and the likelihood of developing AIAA in patients with breast cancer. This finding provides indirect corroboration of the notion that hepatic OATP1B-type transporters regulate concentrations of AIs in plasma, that elevated concentrations of AIs are anticipated in subjects with an impaired uptake transport mechanism in the liver, and that such phenomenon ultimately manifests clinically as an increase in susceptibility to AIAA. The selection of CDCA-24G, a bile acid with high relative abundance in both humans and rodents (50), was based on prior validation studies (30) that identified the agent as a species-independent, highly sensitive and reliable endogenous biomarker of hepatic OATP1B function. The reported lack of increase in CDCA-24G peak plasma concentration and AUC in human subjects homozygous for the *SLCO1B1* c521 T>C variant compared with individuals carrying the reference sequence (51) is likely due to the modest contribution of the this variant to overall hepatic OATP1B function. Irrespective of the mechanistic detail, it should be pointed out that the purpose of our study was not to claim that preclinical findings can be directly translated to clinical findings but merely to provide preliminary support for further exploration of the proposed concept. Indeed, in this rapidly advancing field, additional endogenous substrates of OATP1B, such as the coproporphyrins CP-I and the bile acid glycochenodeoxycholate-3-sulfate, have recently been proposed as validated biomarkers with improved superior selectivity and sensitivity (52). Our future follow-up investigation will integrate an analysis of those potential biomarkers in relation to the relevant drug phenotypes. Furthermore, although patients included in this experiment were randomly selected at a 1:1 ratio for having experienced AIAA or not, the current sample size was relatively small. This suggests that validation of the biomarker data in independent clinical data sets is warranted to corroborate our findings and to further substantiate the reliability of the identified association between hepatic OATP1B-type transporter function and the development of AIAA. Ongoing efforts will also focus on the development and application of scalable physiologically based models to integrate pharmacokinetic, toxicity, and biomarker data from both non-human animals and patients with breast cancer to predict AIAA and allow the development of tailored, individualized dosing strategies that retain activity but lack debilitating side effects.

It is noteworthy that most predictive and/or preventative strategies for AIAA as well as the management of this condition have been shown to be either lacking effectiveness or to be not clinically feasible (53). An alternative approach to address tolerability concerns associated with the therapeutic use of AIs that is presently under intense investigation revolves around recent advancements in clinical trial design and the implementation of intermittent dosing regimens for AIs. An important paradigm shift recently endorsed by both the FDA and the American Society of Clinical Oncology is that intermittent dosing of oral medications can possibly reduce the incidence of severe side effects while retaining potent antitumor efficacy. This strategy has been formally tested for various anticancer agents, including small-molecule kinase inhibitors (54) and AIs. A randomized phase III clinical trial demonstrated that intermittent dosing of exemestane, administered at 25 mg three times a week, was non-inferior to the standard daily dosage of 25 mg in suppressing estrogen levels and reducing breast cancer tissue proliferation (55). Similarly, another randomized phase III study compared continuous treatment with daily letrozole doses of 2.5 mg for 5 years against an intermittent regimen comprising 9 months of daily treatment followed by 3 months off in years 1 to 4 and treating throughout year 5. Results from this trial indicate that 7-year disease-free survival was comparable in both treatment arms although the incidence of arthralgia was only slightly decreased in patients receiving intermittent dosing (56). Findings from both studies showed that in terms of treatment efficacy, intermittent pauses on AI therapy do not seem to affect patient outcomes; however, no obvious advantage with regard to the incidence of arthralgia was observed. The lack of optimal outcomes might be partially attributed to these approaches lacking a mechanism-based, personalized approach to refine dosing for each individual patient. An approach on the basis of a baseline biomarker that serves as a direct surrogate for a mechanism that drives susceptibility to AIAA might suggest a new direction. Particularly advantageous in such biomarker-driven approaches is the ability to provide personalized doses and treatment regimens both before and after the initiation of therapy to mitigate the occurrence of a side effect that is known to impede the patients’ quality of life as well as treatment adherence.

An important final consideration in the context of biomarker implementation to tailor doses of AIs in patients with breast cancer that warrants further investigation is associated with the use of combinatorial regimens in which other anticancer drugs might be given concurrently with AIs. For example, the discovery and development of selective CDK4/6 inhibitors, such as palbociclib, ribociclib, and abemaciclib (56, 57), have resulted in altered management of estrogen receptor–positive breast cancer (56). Importantly, definitive data on the interaction of CDK4/6 inhibitors with OATP1B-type transporters are currently lacking, but it is conceivable that the hepatic uptake of members of this class of drugs is dependent on these proteins and/or has an intrinsic ability to inhibit their function, possibly altering the elimination of AIs and the associated treatment-related arthralgia. Importantly, previous clinical trials have reported an increased incidence of general adverse events in the combination therapies of AIs with CDK4/6 inhibitors in comparison with AI monotherapy (58–61). Further investigations are thus urgently required to shed light on the potential for drug–drug interactions between these two families of drugs and to specifically elucidate the interplay between AIs and CDK4/6 inhibitors.

Collectively, we identified a previously unrecognized pathway of AIAA that is mediated by defects in hepatic OATP1B-type transporters and originates from a downstream cascade involving impaired elimination. These findings shed light on the rate-limiting step in the elimination of AIs and provide a rationale for the prospective implementation of transporter biomarkers associated with OATP1B-type transport function to predict susceptibility to AIAA and ultimately mitigate this debilitating toxicity.

## Supplementary Material

Supplementary documents Supplemental materials

Figure S1 Supplemental figure 1

Figure S2 Supplemental figure 2

Figure S3 Supplemental figure 3

Figure S4 Supplemental figure 4

Figure S5 Supplemental figure 5

Figure S6 Supplemental figure 6
