# Supplementary material for: OATP1B-type Transport Function Is a Determinant of Aromatase Inhibitor–Associated Arthralgia Susceptibility
Source: Cancer Res Commun. 2025 Mar 27;5(3):497–511. doi: 10.1158/2767-9764.CRC-24-0475 (PMC11948302; doi:10.1158/2767-9764.CRC-24-0475)
Supplement: Supplementary documents — Supplemental materials [file crc-24-0475_supplementary_documents_suppsm.docx]

Supplementary Information for

**OATP1B-type transport function is a determinant of aromatase inhibitor-associated arthralgia susceptibility**

Hanieh Taheri^1^, Yang Li^1^, Kevin M. Huang^1^, Eman Ahmed^1^, Yan Jin^1^, Thomas Drabison^1^, Yan Yang^2^, Samuel K. Kulp^1^, Nicholas A. Young^3^, Junan Li^1^, Xiaolin Cheng^2^, Kara N. Corps^4^, Christopher C. Coss^1^, Jennifer E Vaughn^5^, Maryam B. Lustberg^6^, Alex Sparreboom^1^, and Shuiying Hu^1^

**Authors’ Affiliations:** ^1^Division of Pharmaceutics and Pharmacology, and ^2^Division of Medicinal Chemistry and Pharmacognosy, College of Pharmacy, The Ohio State University, Columbus, OH 43210; ^3^Division of Rheumatology and Immunology, Department of Internal Medicine, The Ohio State University Wexner Medical Center, Columbus, OH 43210; ^4^Department of Veterinary Biosciences, College of Veterinary Medicine, The Ohio State University, Columbus, OH 43210; ^5^Division of Hematology, Department of Internal Medicine, The Ohio State University, Columbus, OH 43210; ^6^Yale Comprehensive Cancer Center, Yale School of Medicine, New Haven, CT 06510.

**Corresponding author:** Shuiying Hu, Division of Pharmaceutics and Pharmacology, College of Pharmacy & Comprehensive Cancer Center, The Ohio State University, 496 West 12th Avenue, Columbus, OH 43210. Tel: +1-614-247-6203; Fax: +1-614-688-4028; Email: [hu.1333@osu.edu](mailto:hu.1333@osu.edu)

**Authors’ Disclosures:** MBL has provided consultation to PledPharma and Disarm Therapeutics. The other authors declare no potential conflicts of interest.

**Table S1. Available PDB information for OATP1B1**

|  | **8HNB** | **8HNC** | **8HNH** | **8K6L** | **8HND** | **8PHW** |
| --- | --- | --- | --- | --- | --- | --- |
| Ligand | apo | Bilirubin | Simeprevir | DCF | E3S | E3S |
| State | outward-open | outward-open | outward-open | outward-open | inward-open | inward-open |
| Binding Pocket State | Minor-pocket closed | Minor-pocket closed | Minor-pocket closed | Minor-pocket open | Minor-pocket open | Minor-pocket open |
| Resolution | 3.53 | 3.73 | 3.73 | 2.92 | 3.19 | 3.60 |

Abbreviations: PDB, Protein Data Bank.

**Table S2. Docking scores (kcal/mol) of aromatase inhibitors to Different Conformation States of OATP1B1**

| ­­Drugs | 8HNB^*^ | 8HNC^*^ | 8HNH^*^ | 8K6L^**^ | 8HND^**^ | 8PHW^**^ |
| --- | --- | --- | --- | --- | --- | --- |
| Anastrozole | -7.444 | -4.849 | -7.028 | -5.862 | **-7.634** | -7.324 |
| Letrozole | -6.707 | -6.010 | -6.464 | -6.650 | **-8.295** | -7.695 |
| Exemestane | -5.192 | -6.064 | -6.193 | -5.787 | -7.445 | **-7.634** |

* minor pocket closed states

** minor pocket open states

**Table S3. Docking scores (kcal/mol) of aromatase inhibitors to OATP1B3 Homology Models**

| ­­Drugs | OATP1B3-Model1^*^ | Adjusted-OATP1B3-Model2 ^**^ |
| --- | --- | --- |
| Anastrozole | -5.887 | **-6.132** |
| Letrozole | -5.688 | **-6.575** |
| Exemestane | -5.488 | **-7.380** |

* minor pocket closed states

** minor pocket open states

| **Mouse genotype** | **Treatment (Dose mg/kg)** | **Activ.** | **Stere.** | **Locom.** | **Dist. (m)** | **Res.T. (hr)** | **RT(%)** | **MS(%)** | **MF(%)** |
| --- | --- | --- | --- | --- | --- | --- | --- | --- | --- |
| Wild-type | Vehicle | 40863 | 4162 | 36702 | 538 | 8.88 | 74.07 | 16.37 | 9.57 |
| Wild-type | Letrozole (0.5) | 41163 | 3902 | 37261 | 512 | 9.06 | 75.55 | 15.53 | 8.90 |

**Table S4.**  **Vehicle treatment does not alter the general physical activity of the mice in comparison to the letrozole treatment.**

General activity of the mice following the representative letrozole dosing regimen is summarized in the table (n=3-6 per genotype). Abbreviations: Activ.,Global activity or the sum of the stereotypes and locomotion activities. Stere., Stereotypes is essentially movement without change in position/location. This might include grooming, turning in place, eating, drinking. Locom., is conversely movement with change in position/location. Dist., Distance is the total distance (m) traveled. Res.T, Resting Time is the time period (hr) during which speed is below resting threshold. RT(%), Resting Time (%) is resting time expressed as a % of total duration (12 h). MS(%), Moving Slow (%) is moving slow time expressed as a % of total duration (12 h). MF(%), Moving Fast (%) is moving fast time expressed as a % of total duration (12 h). Movement thresholds are as follows: <2.0 cm/s = Resting; >2.0 cm/s and <5.0 cm/s = Slow movement; >5.0 cm/s = Fast movement.

**Table S5. Summary of 20 breast cancer patients’ information.**

| **Patient ID** | **Age of dx (Y)** | **Radiation history** | **Treatment history** | **Treatment duration** | **Arthralgia symptoms** | **CDCA-24G baseline plasma concentration (ng/ml)** |
| --- | --- | --- | --- | --- | --- | --- |
| 1 | 65 | Yes | Tamox, Exe, Rised | 2Y, 5Y, 3Y | No | 0.29 |
| 2 | 59 | Yes | Exem, Alen | 4.5Y, N/A | No | <LLOQ |
| 3 | 61 | Yes | Ana, Rised | 5Y, 5Y | No | 0.93 |
| 4 | 63 | No | Tamox, Anas | 5Y, N/A | No | 2.11 |
| 5 | 77 | Yes | Ana | 2Y | No | 4.10 |
| 6 | 75 | Yes | Ana, Rised | 6Y, 3.5Y | No | 0.60 |
| 7 | 71 | Yes | Ana | 5Y | No | 2.31 |
| 8 | 50 | Yes | Ana, Let, Exe | 4Y, 2M, N/A | No | 0.21 |
| 9 | 57 | Yes | Ana | 3Y | No | 0.025 |
| 10 | 69 | Yes | Tamox, Ana, Alen, | 5Y, 5Y, N/A | No | 2.75 |
| 11 | 87 | No | Ana, Let, Exem | 0.5Y, N/A, <1Y | Yes | 5.06 |
| 12 | 62 | Yes | Let | N/A | Yes | 3.61 |
| 13 | 75 | Yes | Ana | 5Y | Yes | 3.99 |
| 14 | 45 | Yes | Gose, Ana, Let | N/A, 1Y, N/A | Yes | 3.32 |
| 15 | 55 | Yes | Let, Exem, Ana | 9M,4Y, 5Y | Yes | 10.9 |
| 16 | 41 | No | Tamox, Exem, Let | 3Y, <1Y, 3Y | Yes | 3.67 |
| 17 | 67 | No | Let | 1Y | Yes | 11.9 |
| 18 | 65 | No | Ana | 3Y | Yes | 3.29 |
| 19 | 58 | Yes | Let, Alen | 2.5Y, N/A | Yes | 7.03 |
| 20 | 83 | No | Ana, Tamox | 2Y, N/A | Yes | 3.68 |

Abbreviations: Ana, Anastrozole; Let, Letrozole; Exem, Exemestane; Tamox, Tamoxifen; Rised, RISEDRONATE; Alen, ALENDRONATE; Gose, Goserlin; Y,Year; M,Month; LLOQ, Lower Limit Of Quantification.

Drugs families: Aromatase inhibitors: Ana, Let, Exe; Estrogen receptor modulator: Tamox; Bisphosphonate: Rised, Alen; Gonadotropin-releasing hormone: Gose.
