# Supplementary figures and images for: OATP1B-type Transport Function Is a Determinant of Aromatase Inhibitor–Associated Arthralgia Susceptibility
Source: Cancer Res Commun. 2025 Mar 27;5(3):497–511. doi: 10.1158/2767-9764.CRC-24-0475 (PMC11948302; doi:10.1158/2767-9764.CRC-24-0475)

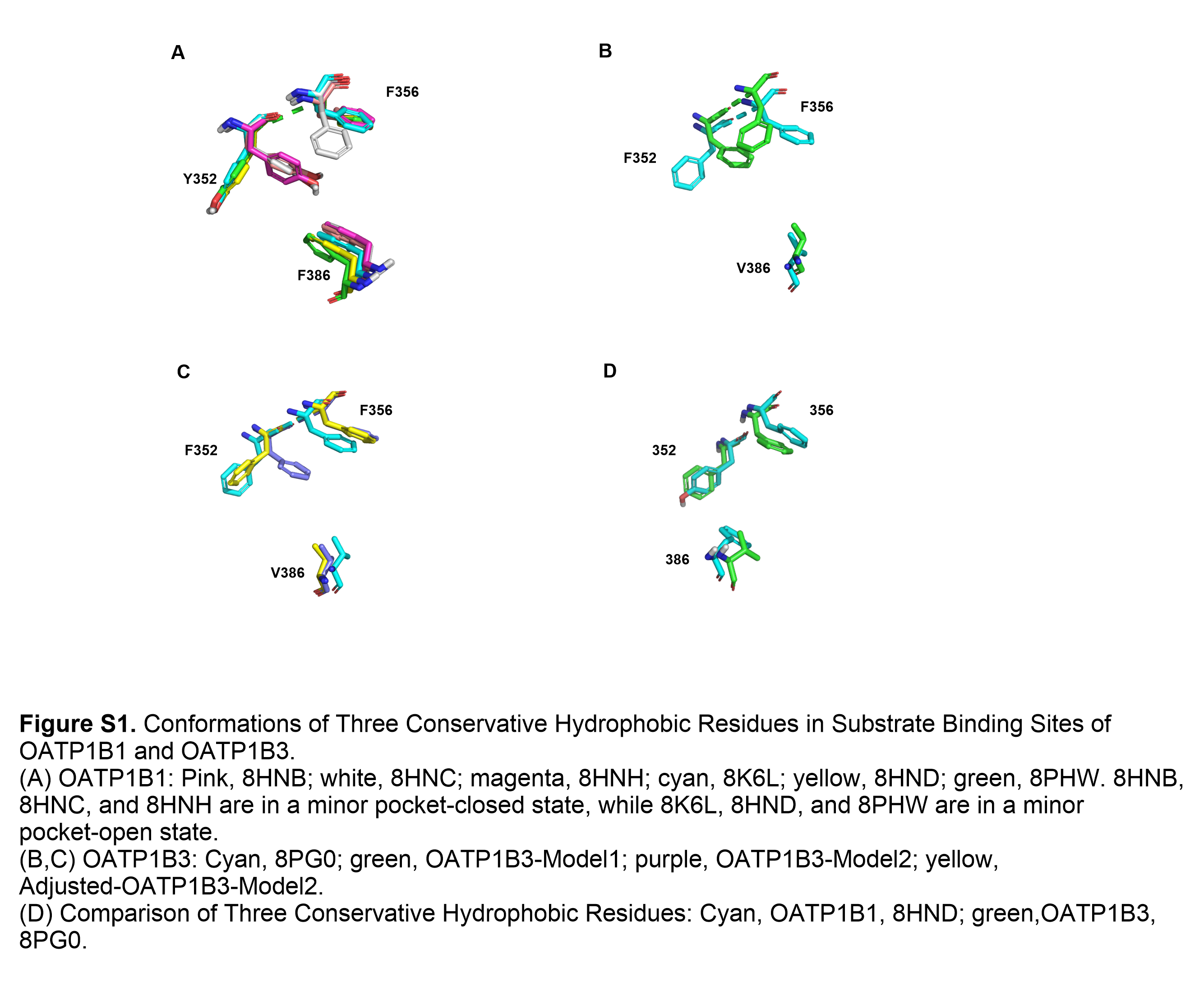

Supplement: Figure S1 — Supplemental figure 1 [file crc-24-0475_figure_s1_suppsf1.png]

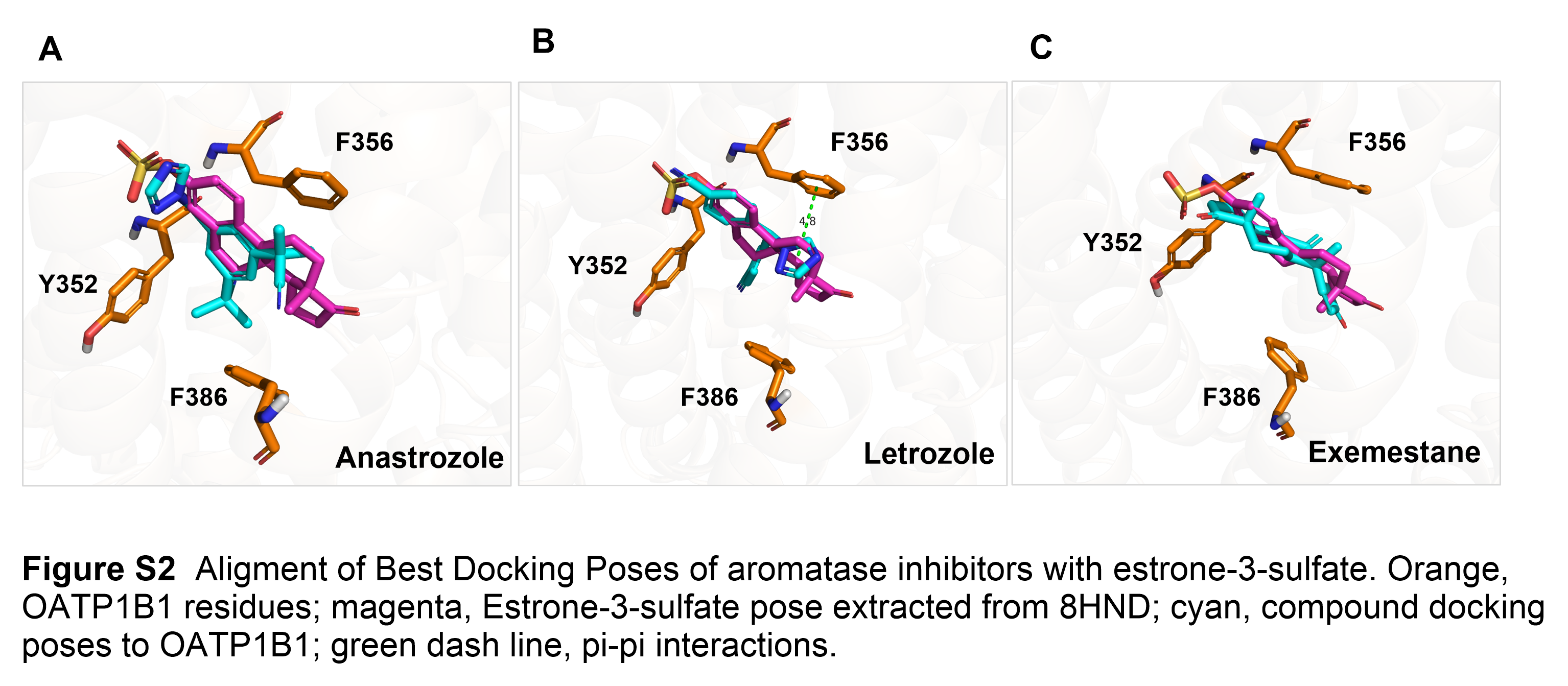

Supplement: Figure S2 — Supplemental figure 2 [file crc-24-0475_figure_s2_suppsf2.png]

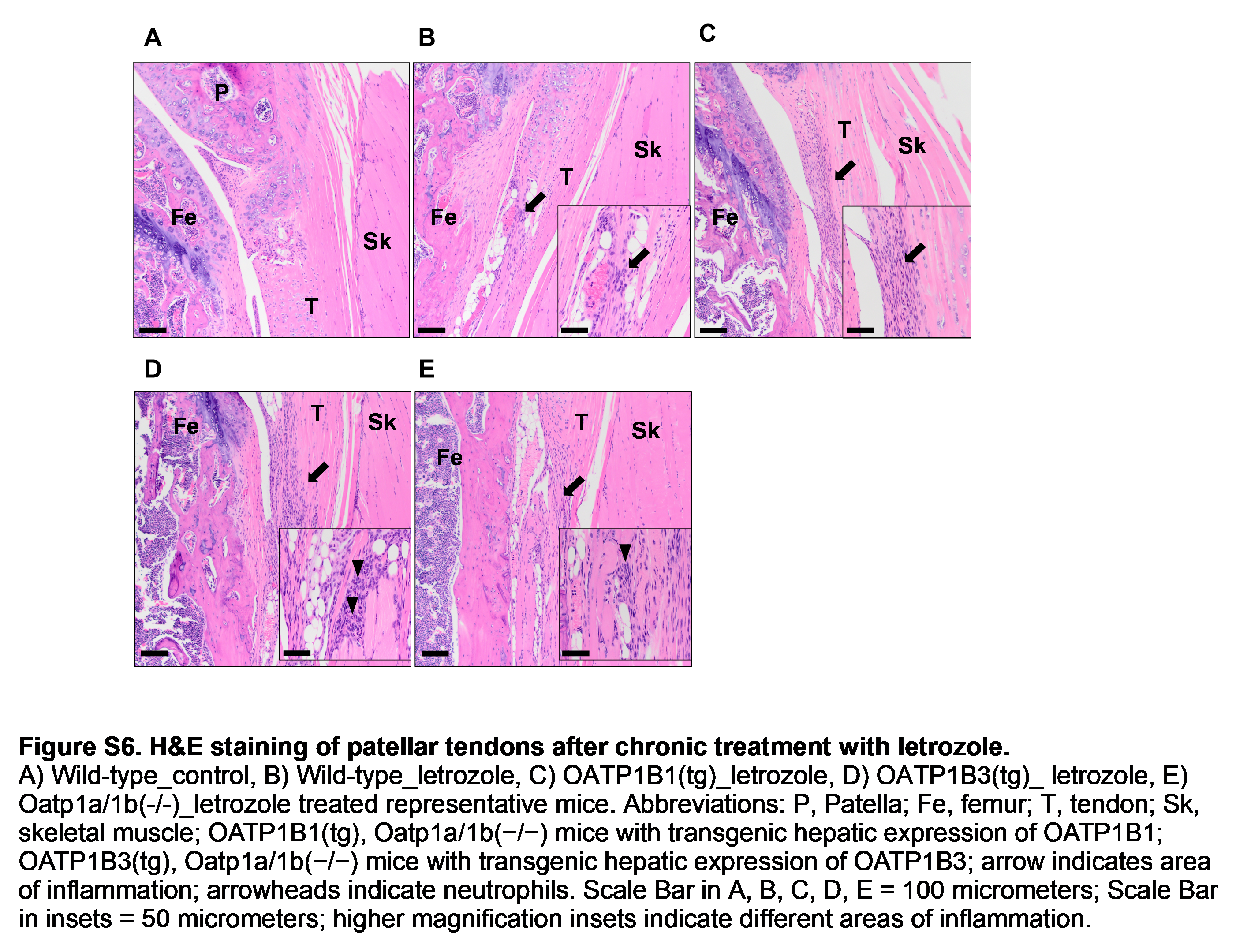

Supplement: Figure S6 — Supplemental figure 6 [file crc-24-0475_figure_s6_suppsf6.png]
