# Supplementary material for: OATP1B-type Transport Function Is a Determinant of Aromatase Inhibitor–Associated Arthralgia Susceptibility
Source: Cancer Res Commun. 2025 Mar 27;5(3):497–511. doi: 10.1158/2767-9764.CRC-24-0475 (PMC11948302; doi:10.1158/2767-9764.CRC-24-0475)
Supplement: Figure S3 — Supplemental figure 3 [file crc-24-0475_figure_s3_suppsf3.pptx]

## Slide 1
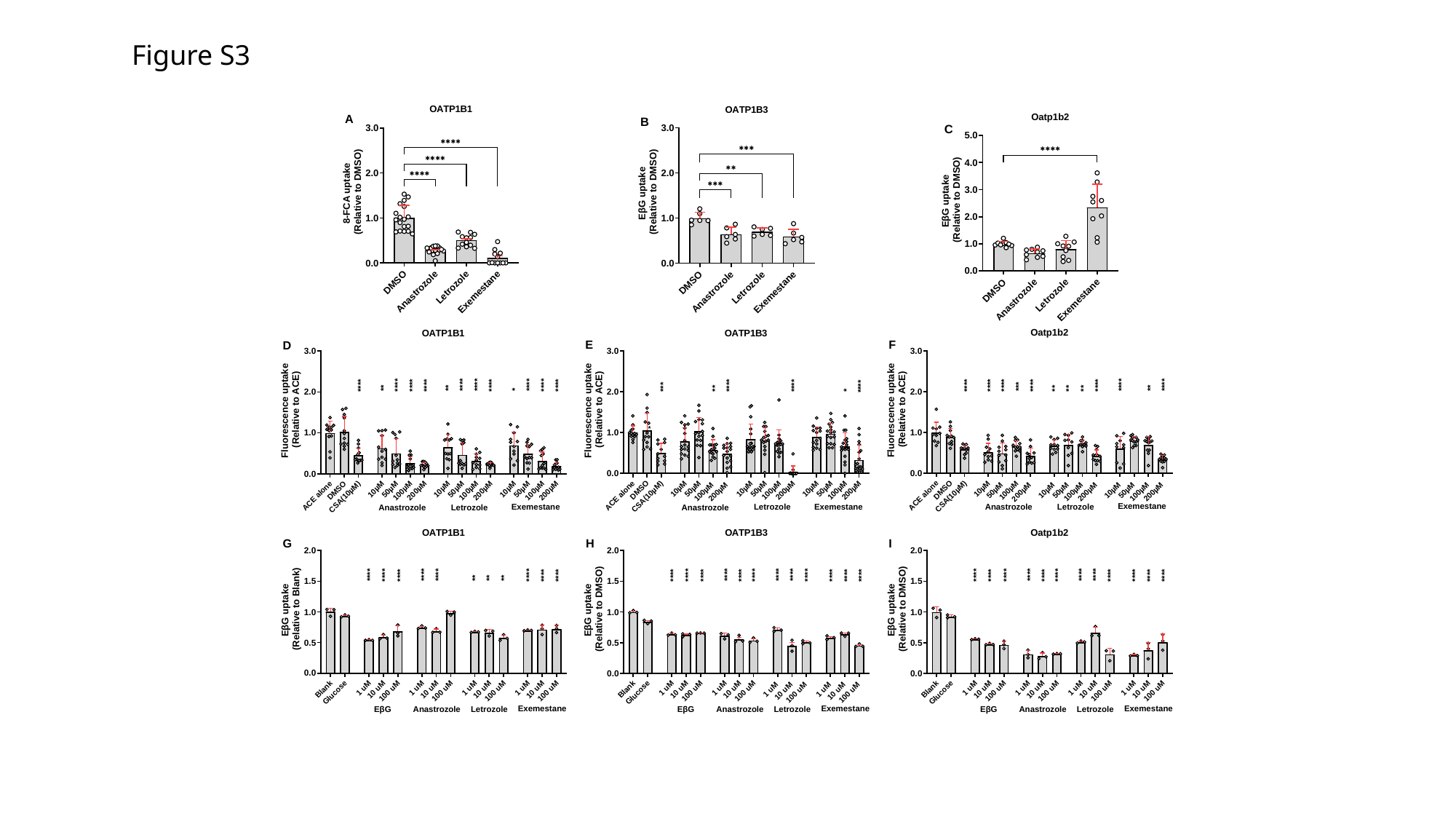

Figure S3

## Slide 2
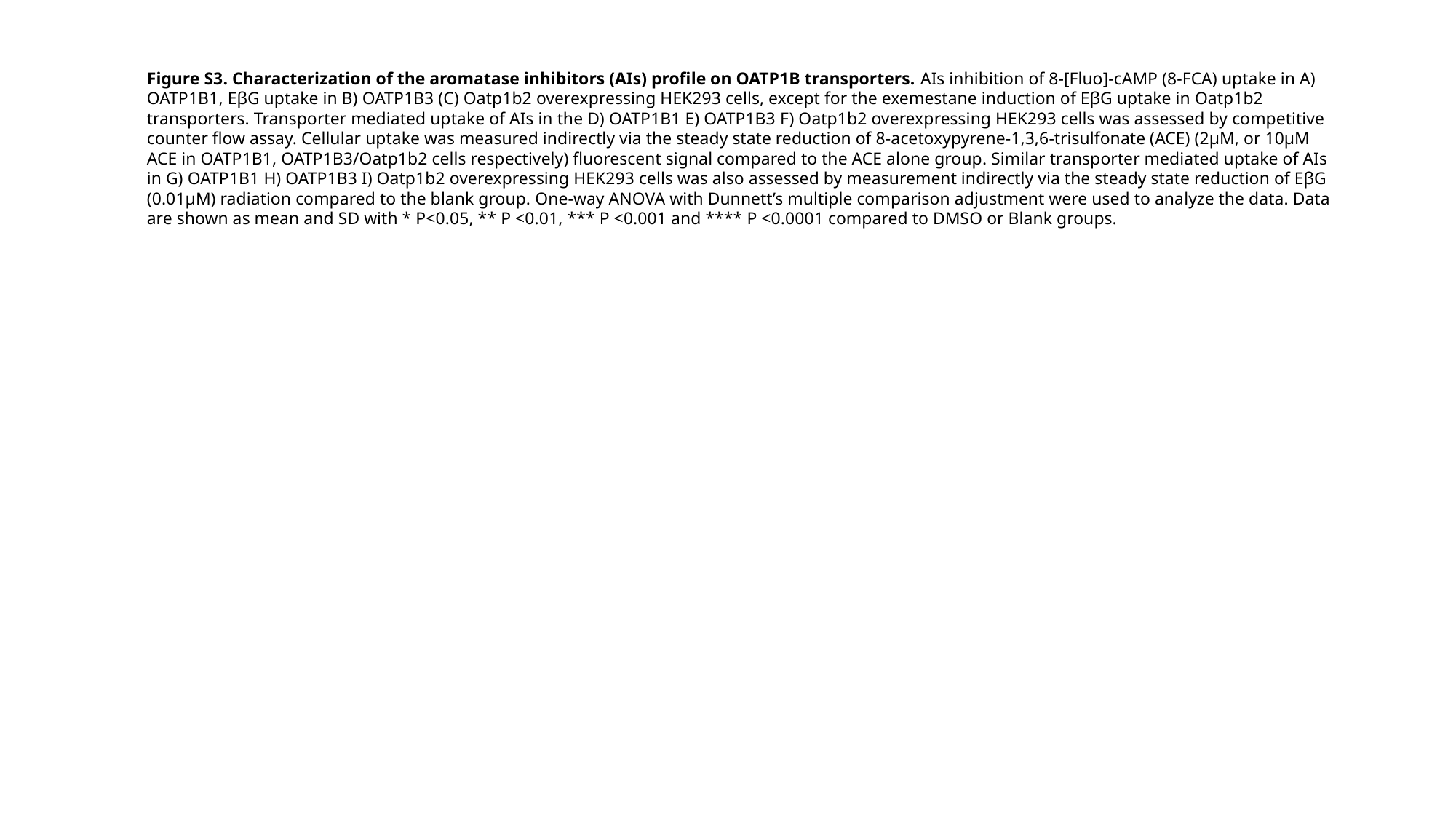

Figure S3. Characterization of the aromatase inhibitors (AIs) profile on OATP1B transporters. AIs inhibition of 8-[Fluo]-cAMP (8-FCA) uptake in A) OATP1B1, EβG uptake in B) OATP1B3 (C) Oatp1b2 overexpressing HEK293 cells, except for the exemestane induction of EβG uptake in Oatp1b2 transporters. Transporter mediated uptake of AIs in the D) OATP1B1 E) OATP1B3 F) Oatp1b2 overexpressing HEK293 cells was assessed by competitive counter flow assay. Cellular uptake was measured indirectly via the steady state reduction of 8-acetoxypyrene-1,3,6-trisulfonate (ACE) (2μM, or 10µM ACE in OATP1B1, OATP1B3/Oatp1b2 cells respectively) fluorescent signal compared to the ACE alone group. Similar transporter mediated uptake of AIs in G) OATP1B1 H) OATP1B3 I) Oatp1b2 overexpressing HEK293 cells was also assessed by measurement indirectly via the steady state reduction of EβG (0.01μM) radiation compared to the blank group. One-way ANOVA with Dunnett’s multiple comparison adjustment were used to analyze the data. Data are shown as mean and SD with * P<0.05, ** P <0.01, *** P <0.001 and **** P <0.0001 compared to DMSO or Blank groups.
