# Supplementary material for: OATP1B-type Transport Function Is a Determinant of Aromatase Inhibitor–Associated Arthralgia Susceptibility
Source: Cancer Res Commun. 2025 Mar 27;5(3):497–511. doi: 10.1158/2767-9764.CRC-24-0475 (PMC11948302; doi:10.1158/2767-9764.CRC-24-0475)
Supplement: Figure S4 — Supplemental figure 4 [file crc-24-0475_figure_s4_suppsf4.pptx]

## Slide 1
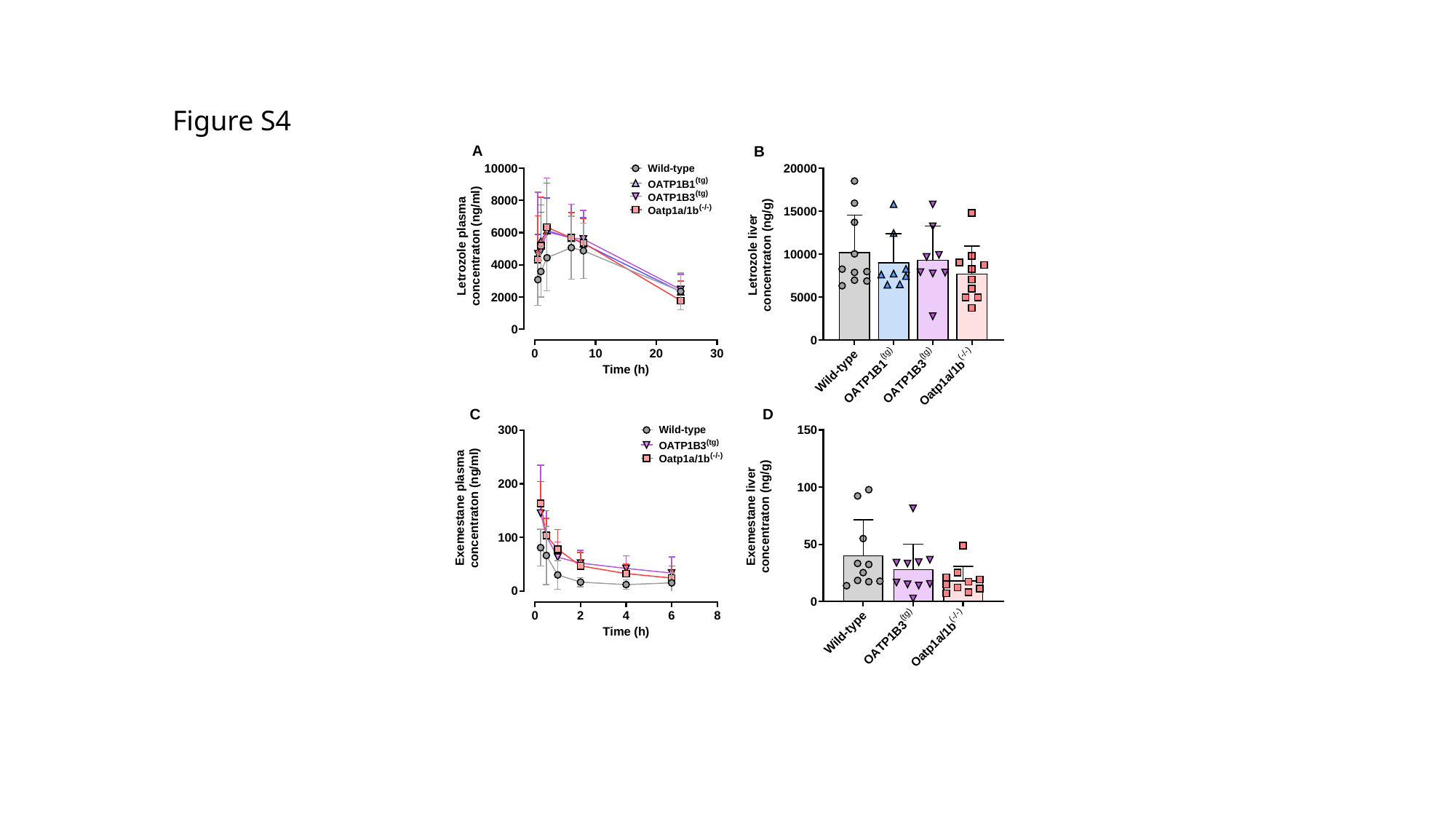

Figure S4

## Slide 2
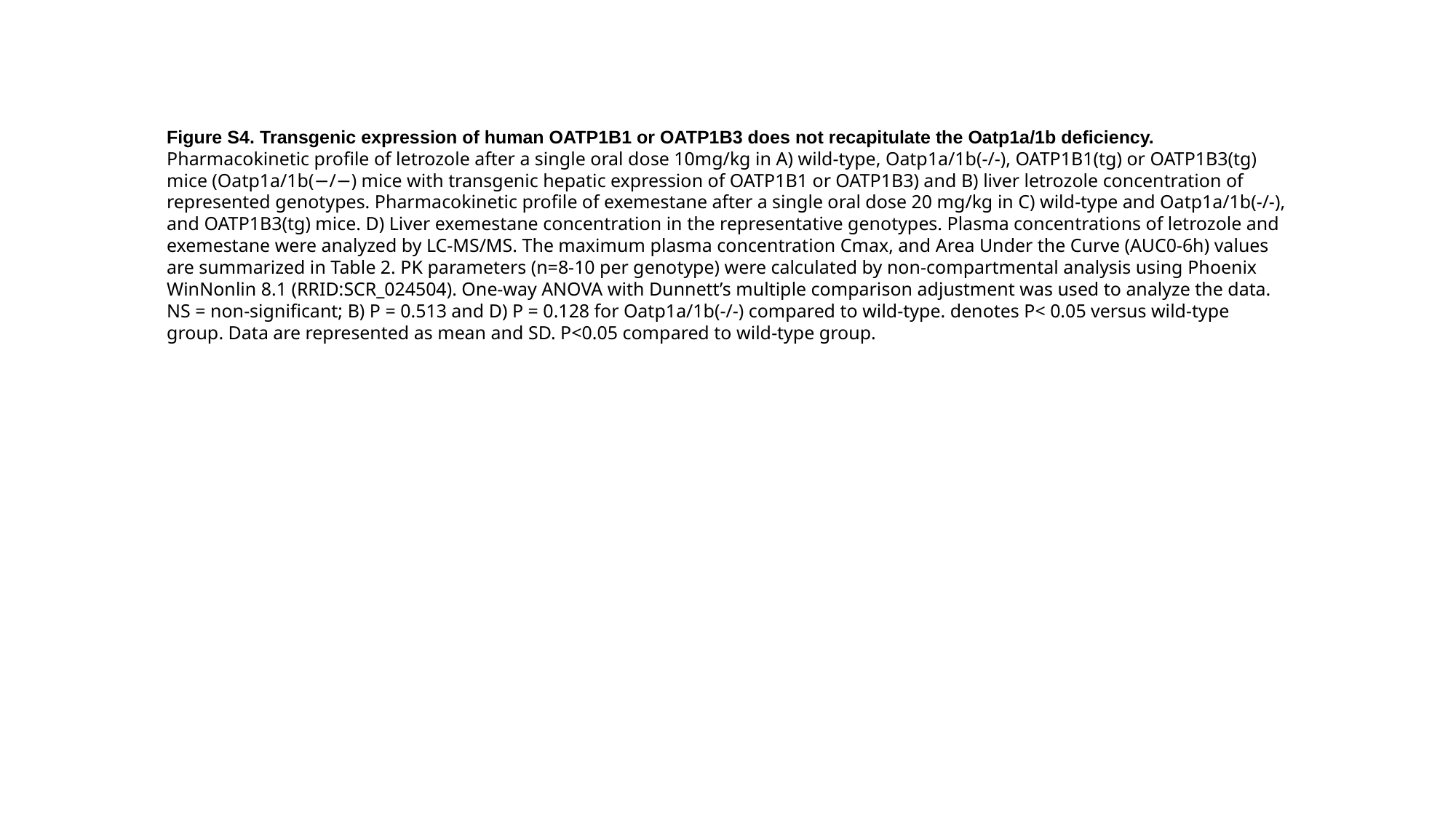

Figure S4. Transgenic expression of human OATP1B1 or OATP1B3 does not recapitulate the Oatp1a/1b deficiency. Pharmacokinetic profile of letrozole after a single oral dose 10mg/kg in A) wild-type, Oatp1a/1b(-/-), OATP1B1(tg) or OATP1B3(tg) mice (Oatp1a/1b(−/−) mice with transgenic hepatic expression of OATP1B1 or OATP1B3) and B) liver letrozole concentration of represented genotypes. Pharmacokinetic profile of exemestane after a single oral dose 20 mg/kg in C) wild-type and Oatp1a/1b(-/-), and OATP1B3(tg) mice. D) Liver exemestane concentration in the representative genotypes. Plasma concentrations of letrozole and exemestane were analyzed by LC-MS/MS. The maximum plasma concentration Cmax, and Area Under the Curve (AUC0-6h) values are summarized in Table 2. PK parameters (n=8-10 per genotype) were calculated by non-compartmental analysis using Phoenix WinNonlin 8.1 (RRID:SCR_024504). One-way ANOVA with Dunnett’s multiple comparison adjustment was used to analyze the data. NS = non-significant; B) P = 0.513 and D) P = 0.128 for Oatp1a/1b(-/-) compared to wild-type. denotes P< 0.05 versus wild-type group. Data are represented as mean and SD. P<0.05 compared to wild-type group.
