# Supplementary material for: OATP1B-type Transport Function Is a Determinant of Aromatase Inhibitor–Associated Arthralgia Susceptibility
Source: Cancer Res Commun. 2025 Mar 27;5(3):497–511. doi: 10.1158/2767-9764.CRC-24-0475 (PMC11948302; doi:10.1158/2767-9764.CRC-24-0475)
Supplement: Figure S5 — Supplemental figure 5 [file crc-24-0475_figure_s5_suppsf5.pptx]

## Slide 1
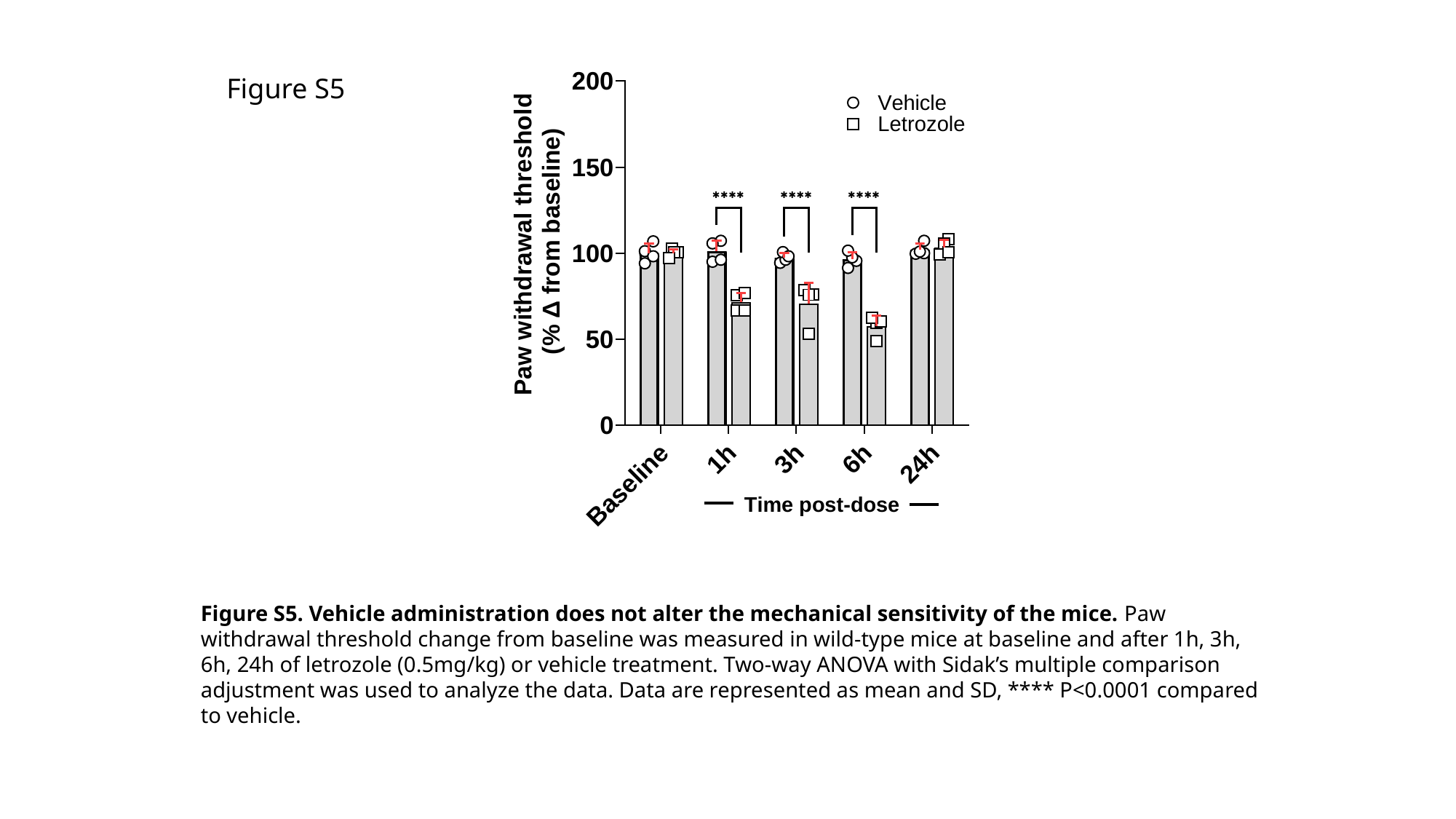

Figure S5
Figure S5. Vehicle administration does not alter the mechanical sensitivity of the mice. Paw withdrawal threshold change from baseline was measured in wild-type mice at baseline and after 1h, 3h, 6h, 24h of letrozole (0.5mg/kg) or vehicle treatment. Two-way ANOVA with Sidak’s multiple comparison adjustment was used to analyze the data. Data are represented as mean and SD, **** P<0.0001 compared to vehicle.
